# Probing rock rupture with naturally occurring nuclide signals

**DOI:** 10.1073/pnas.2602434123

**Published:** 2026-04-09

**Authors:** Jia-Qing Zhou, Rong Mao, Xin Luo, M. Bayani Cardenas, Yi-Feng Chen, Fu-Shuo Gan, Chuang-Bing Zhou, Changdong Li, Huiming Tang, Ran Hu, Zhibing Yang, Michael Manga

**Affiliations:** ^a^State Key Laboratory of Water Resources Engineering and Management, Wuhan University, Wuhan 430072, China; ^b^Laboratory for Sea Space Agent, Department of Earth and Planetary Sciences, The University of Hong Kong, Hong Kong 999077, China; ^c^Key Laboratory of Rock Mechanics in Hydraulic Structural Engineering of the Ministry of Education, Wuhan University, Wuhan 430072, China; ^d^Center for Natural Resources, Department of Civil and Environmental Engineering, New Jersey Institute of Technology, Newark, NY 07102; ^e^Department of Earth and Planetary Sciences, The University of Texas at Austin, Austin, TX 787812; ^f^Faculty of Engineering, China University of Geosciences, Wuhan 430074, China; ^g^Department of Earth and Planetary Science, University of California Berkeley, Berkeley, CA 94720

**Keywords:** nuclides, geohazards, precursors, rock failure

## Abstract

Rocks release subtle geochemical warning signals before breaking. These signals, coming from naturally occurring nuclides (e.g., radon, helium, argon, and thoron), have often been reported before earthquakes, volcanic eruptions, landslides, and rock and ice avalanches. However, despite their high sensitivity to deformation, their detectability, as well as myriad promising observations over half a century, nuclide signals are still far from being applied to geohazard prediction or widely used for monitoring. Here, we first develop a decomposition and interpretation method for nuclide signals. By analyzing nuclide signal time series observed from a month-long laboratory rock failure experiment and year-long slope deformation in a field setting, we identify a universal paradigm unit of nuclide signal evolution. We find that this paradigm unit is characterized by two core characteristics: a transient pulse and equilibrium fluctuation which are intrinsically correlated to rupture area and crack aperture, respectively. Through analytical derivation and pore-scale simulations, we establish the constitutive equations that link these characteristic nuclide signals to key rupture structural parameters. Rooted in these constitutive relations, we further develop a diagnostic theory of rock rupture via nuclide signals. We apply the model to track rock failures at the laboratory and field scale. The proposed nuclide signal decomposition and rupturing model enable the unification of discrete signal units emitted by individual microrupturing events, with the integrated signal evolution observed during macroscopic failure. This integration may serve as a foundation for both the mesoscopic assessment of rock damage and the early warning of geohazards induced by rock ruptures.

## Significance

The idea of hydrogeochemical precursors for geological hazards such as earthquakes, volcanic eruptions, and landslides has been proposed over half a century. Numerous lab experiments and field observations, especially on naturally occurring nuclides, have proposed a link between nuclide anomalies and the occurrence of geohazards. Moving beyond conceptual ideas and empirical observations requires a physical model to connect measurements to process. We develop a model to decode the critical failure states of rock rupture via two measured nuclide signals. Our work removes a key theoretical barrier to the long-sought goal of nuclide-based rock rupture prediction, paving the way for better early warning of geohazards and rock engineering management.

Predicting the rupture of subsurface rocks is a central challenge across the Earth sciences, with considerable practical importance for forecasting geohazards, such as earthquakes, volcanic eruptions, landslides, and avalanches. Despite decades of research and investment, such rupturing events remain difficult to anticipate, as they originate below the surface, beyond the reach of direct observation. A long-standing hope is to identify reliable precursors that could signal impending rock ruptures. Among these, the release of naturally occurring nuclides (e.g., ^222^Rn, ^4^He, ^40^Ar, thoron) from stressed and breaking rocks has been of particular interest owing to the combination of sensitivity to structural alteration and practical detectability, as these signals originate at depth but escape to the surface (1–8).

Field monitoring has sometimes revealed striking nuclide anomalies, often orders of magnitude above background levels, preceding major rupturing events (1, 3–5, 9–19). With advances in sensor technology, we now possess an abundance of high-resolution temporal signals, capturing these precursory phenomena in unprecedented detail. Yet, the transition from retrospective documentation to practical prediction remains an open challenge. This inability stems in part from limitations in the theoretical models that underpin the origin of the signals, and existing efforts remain largely phenomenological, whether based on nuclide emanation (2–5, 20, 21) and transmission characterization (22, 23), or spectral analysis of nuclide signals (24, 25). Empirical approaches describe, but do not explain physically, the couplings between mechanical processes of rock ruptures and the genesis, transmission, and amplification of nuclide signals. Absent a mechanistic constitutive formulation that connects hydro-chemo-mechanical couplings, signals cannot be quantitatively inverted to forecast rupture.

Here we derive a paradigm unit of nuclide signal evolution by decomposing radon anomaly time series into features produced by distinct processes, and establish a model that rigorously couples rupture-induced structural alterations to characteristic signals. Unlike previous hindcast interpretations, this theory provides a physical basis to invert temporal nuclide anomalies into quantitative diagnoses of changes that precede rupture. Assessed across scales, this framework offers a path from taking nuclide monitoring as a descriptive tool to an approach for quantifying changes in rock mechanical properties, with promise to help unlock the potential held by decades of precursor observations.

## Results

### Physical and Observational Basis for Probing Rock Rupture via Nuclide Signals.

**Fig. 1.**
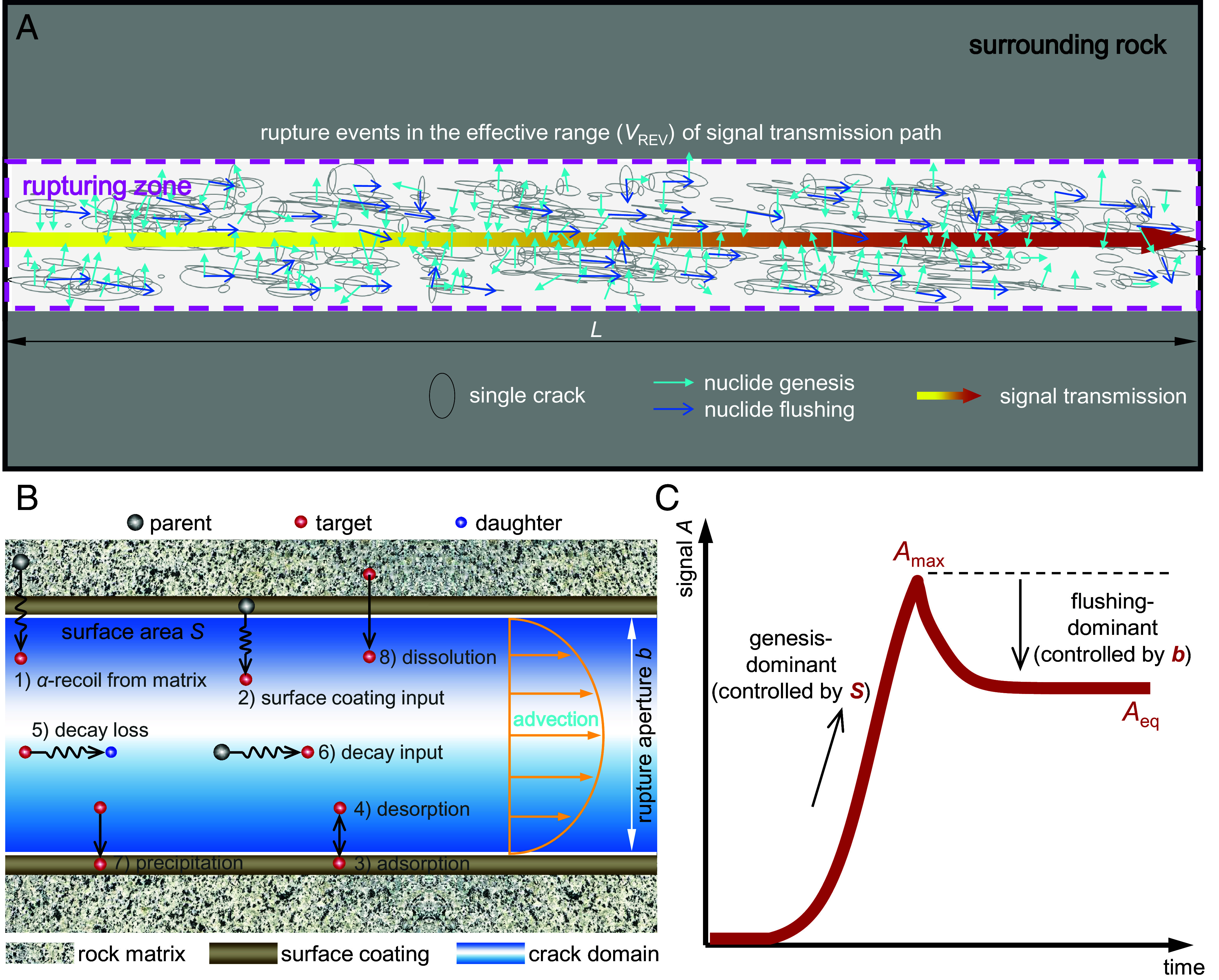
Conceptualization of using characteristic nuclide signals to probe rock rupture. (*A*) Illustration of rock rupture events and accompanying nuclide signals. Macroscopic rock rupture consists of a series of microrupturing events, as represented by a cluster of single cracks. These events collectively form a dominant rupture direction, which coincides with the transmission direction of associated nuclide signals (the length of transmission path, *L*). As such, nuclide signals can track rupturing events along their transmission path, with the Volume Representative Elementary Volume (*V*_REV_, marked by the pink dashed box in the figure) defined as the corresponding rupturing domain. (*B*) All possible physicochemical processes and key structural parameters (rupture area *S* and aperture *b*) affecting nuclide signal transmission in a single rupturing crack. *S* governs the nuclide emanation process within the crack domain, while *b* dominates the flow regime in this domain and thus controls the nuclide flushing process. (*C*) Inferred paradigm unit of nuclide signals, incorporating the control of key characteristic signal intensities and their corresponding structural parameters. The paradigm unit exhibits a distinct evolution trajectory: an initial surface area (*S*) controlled phase dominated by nuclide genesis, manifesting as a transient pulse peaking at *A*_max_, followed by a crack aperture (*b*) controlled flushing phase during the advection–diffusion process. This latter stage drives signal attenuation until equilibrium (*A*_eq_) is achieved. The characteristic curve in C is mathematically defined by a piecewise asymmetric logistic function (see Eq. **2a** in M&M).

Rock rupture across multiple scales, ranging from laboratory-scale damage to field-scale fault displacements and rock mass instabilities, is the culmination of the spatiotemporal superposition of microrupturing events (Fig. 1*A*). These microrupturing events generate a series of cracks, from which naturally occurring nuclide signals are released and converge along the dominant rupture direction to form a signal transmission pathway. For each newly initiated crack, nuclides therein undergo a sequence of physicochemical processes (26–28): first, recoil from parent nuclide decay within the rock matrix or surface coating (processes 1 and 2), followed by a series of adsorption/desorption reactions (processes 3 and 4), decay loss and parental decay input (processes 5 and 6), precipitation and dissolution (processes 7 and 8), as well as diffusion and advection (Fig. 1*B*). Among these processes, processes 1 and 2 dominate nuclide genesis and are governed by the rupture area *S* (2, 22, 26). The advection process controls the nuclide flushing process, which is regulated by the rupture aperture *b* (29). During crack initiation, a sudden increase in *S* induces a sharp rise in the nuclide genesis rate, resulting in a rapid surge in signal intensity. During the subsequent dilation and closure of the new crack, variations in *b* alter the flushing rate, causing the signal to eventually stabilize with varying attenuation magnitudes and rates. Therefore, the detected nuclide signal (*A*) is determined by the interplay between the genesis and the flushing rates.

Our diagnostic theory is built upon the hypothesis that the evolution of any nuclide signal triggered by a microrupturing event follows a characteristic trajectory (Fig. 1*C*). Specifically, the signal initiates with a rapid, transient release from rock or grain surfaces (controlled by crack surface area *S*), manifesting as a transient pulse that peaks at *A*_max_. As the signal enters the transmission pathway, it undergoes attenuation driven by flushing (controlled by crack aperture *b*) and physicochemical reactions, eventually reaching an equilibrium state (*A*_eq_). Therefore, the life cycle of any nuclide signal emitted from rock rupture can be defined by the two key parameters *A*_max_ and *A*_eq_. The characteristic curve presented in Fig. 1*C* thus serves as the paradigm unit for nuclide signal evolution during rock rupture (the functional form of this unit is provided in *Section 1* of M&M).

**Fig. 2.**
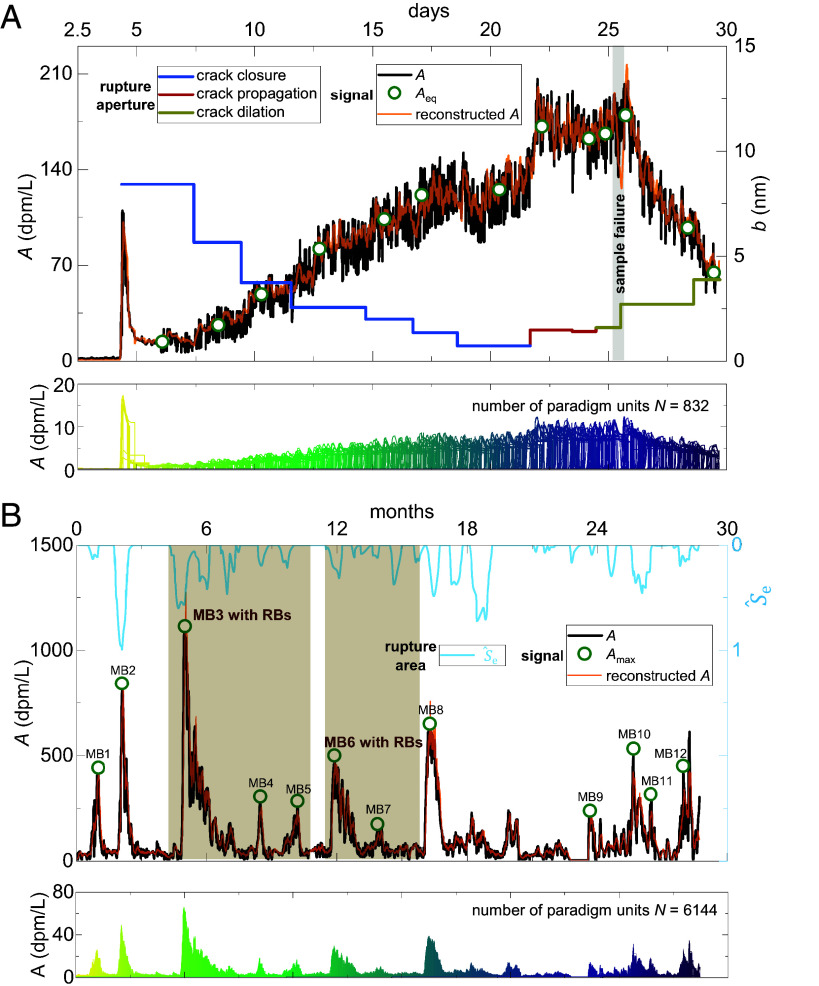
Decomposition and reconstruction of observed radon signal (dpm⋅L^−1^ where dpm denotes disintegrations per minute) time series during rock ruptures across different scales. (*A*) Interpretation of month-long laboratory observations of radon signal evolution for a stressed crustal granite (2). The signal exhibits periodic and phased evolutionary characteristics, caused by crack initiation & opening, crack dilation & closure, or crack further propagation. These transitions are corroborated by the equivalent rupture apertures calculated from measured permeability. Under any given stress condition (i.e., a specific crack aperture *b*), the nuclide signal reaches an equilibrium *A*_eq_, identified by the green circles. The entire month-long radon signal time series can be effectively decomposed into 832 paradigm units (illustrated at the bottom of *A*) of varying intensities and durations (*Section 1* of M&M for decomposition method; *SI Appendix*, Fig. S1 for error estimation). (*B*) Interpretation of year-long observations of radon bursts from hillslope bedrock triggered by periodic reservoir impoundment (20). Approximately 12 main radon bursts (MBs) were observed, with their transient pulses ${\hat{A}}_{max}$ identified by green circles. Each MB is followed by a succession of moderate or minor residual bursts (RBs). During the monitoring period, the apparent rupture area (${\hat{S}}_{e}$) was derived concurrently. This year-long radon signal time series can be effectively reconstructed through the integration of 6,144 paradigm units (illustrated at the bottom of *B*) with diverse intensities and periods (*Section 1* of M&M; *SI Appendix*, Fig. S2 for error estimation).

The detected nuclide signals in real scenarios are formed by the spatial and temporal superposition of a series of paradigm units. To verify the existence of such paradigm units and the validity of the proposed basis function, we develop a decomposition method for nuclide signal time series (see details of the method in *Section 1* of M&M and *SI Appendix, Text S1*). From the literature, two classic long-term radon (^222^Rn) signal datasets recorded during rock rupture processes were selected for analysis: one from month-long laboratory observations of radon signals in stressed crustal granite (2), and the other from year-long field observations of radon bursts in hillslope bedrock triggered by periodic reservoir impoundment (20). Analysis results demonstrate that the radon signal sequences recorded during laboratory rock failure (Fig. 2*A*) and reservoir-induced hillslope deformation (Fig. 2*B*) can be decomposed into a set of paradigm units. Specifically, the laboratory and field datasets are characterized by 832 and 6,144 paradigm units with varying intensities and durations, respectively, yielding high coefficients of determination (R^2^ = 0.96 and 0.87). We thus propose that regardless of the scale, intensity, or density of rock ruptures, the underlying nuclide signal evolution intrinsically follows the pattern defined by *A*_max_ and *A*_eq_ with complexities arising from the superposition of paradigm units. This universality provides a physical basis for establishing constitutive relationships between rock geomechanical properties, their evolution, and nuclide signal signatures.

It is important to clarify that the applicability of the paradigm unit to different nuclides is contingent on the specific application context. The unit characterizes nuclide signals from two complementary features of rupture. One feature links *A*_max_ to the rupture area. In this context, the paradigm unit is mainly applicable to natural nuclides hosted in rock minerals, including members of the U-Th decay series (such as Ra~Rn and ^4^He) and the ^40^K ~ ^40^Ar decay system. These nuclides are produced by the decay of parent nuclides within mineral lattices and released via recoil, making them direct proxies for rupture area. The other feature connects *A*_eq_ to rupture aperture. It relies on the principle that aperture changes modulate the fluid velocity field, thereby driving variations in tracer transport. This framework is broadly applicable to mobile tracers, including stable nuclides, radionuclides, and artificial ones. Notably, the practical utility of each nuclide is governed by intrinsic properties such as origin, half-life, mobility, and detectability. For example, radon is highly versatile for near-surface applications (e.g., fault mapping, landslide monitoring, and earthquake precursor detection) due to its moderate half-life, high mobility, and exceptional detectability in shallow environments (26, 27, 30). In contrast, helium is well suited for characterizing deep crustal and mantle processes, such as volcanic systems and deep fault zones (5, 7). Argon, meanwhile, serves as an effective tracer for long-term crustal deformation and paleofluid pathways, as its accumulation within rock matrices records cumulative deformation over geologic time scales (31, 32).

### Constitutive Relations between Signals and Rupture.

**Fig. 3.**
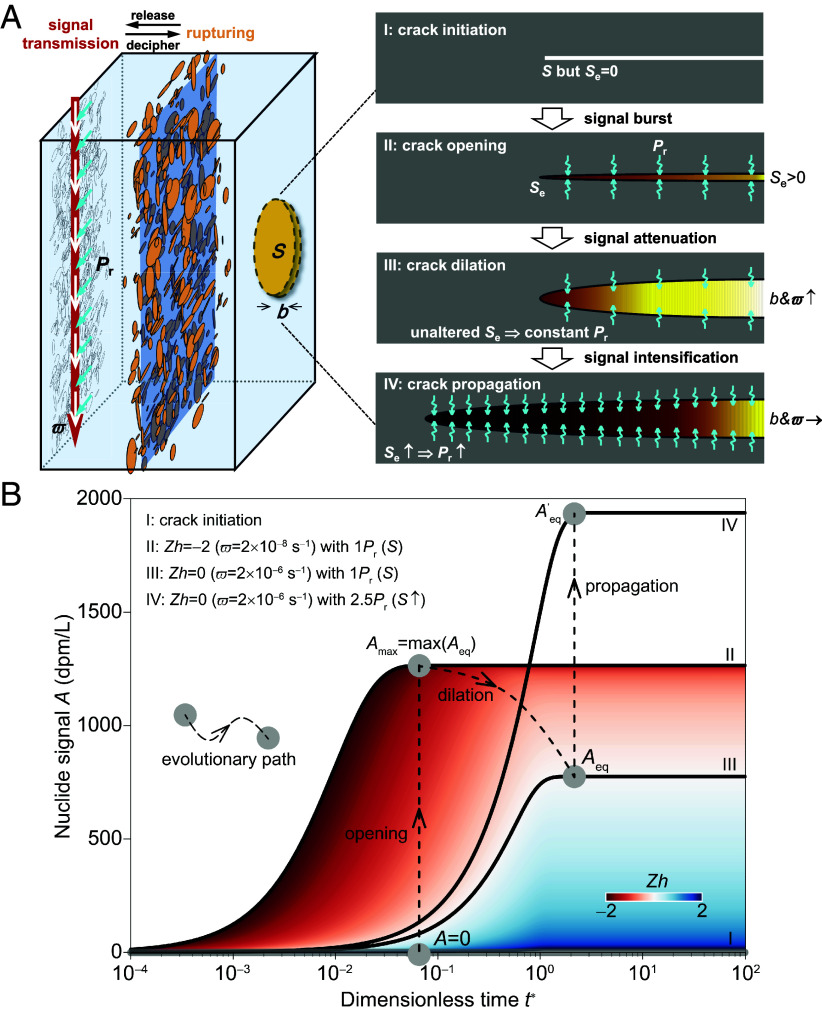
Physical model and analytical implementation of nuclide signal responses to rock rupture. (*A*) Transmission of nuclide signals during a series of single rupture events can be upscaled to a one-dimensional (1D) model. In this model, an increase in crack intensity (quantified by exposed surface area *S*_e_) is equivalent to an increase in transverse uniform recoil rate (*P*_r_) input, while the expansion of the crack system volume (quantified by *b*) is represented by a rise in the longitudinal flushing rate (*ϖ*) (*SI Appendix*, Fig. S3). *ϖ* is defined as the ratio of flow rate to crack volume, and its variation is indicative of changes in *b*. The representative evolution of a single rock rupture progresses through four stages: crack initiation from intact rock or complete closure of preexisting crack (I), crack opening (II), crack dilation (III), and crack propagation (IV). The released signals are intrinsically controlled by two key structural rupture parameters: *b* and *S*_e_, via their impacts on *ϖ* and *P*_r_. (*B*) Theoretical evolutionary pathways of nuclide signals in response to rock rupturing states. Dimensionless time (t^*^) is defined as *tq*/*bL*, where *L* is the signal transmission length and *q* is the specific flow rate. The dimensionless number *Zh* = log (*ϖ*/*λ*) normalizes *ϖ* by the nuclide decay rate (*λ*). During crack opening (state II), *A*_eq_ reaches its maximum (i.e., *A*_max_) across all dilation states (III). Following crack propagation (state IV), the signal rises to a new equilibrium level, characterized by an updated *A*_eq_ and *A*_max_ (when *ϖ* ≈ 0). Results in the figure are derived from radon-specific analytical solutions (*Section 2* of M&M) and are benchmarked against pore-scale numerical simulations that model a range of nuclides, including radon (*SI Appendix*, Figs. S9 and S10).

By quantitatively relating *A*_max_ and *A*_eq_ with the key rupturing stages, we can establish the constitutive relations between nuclide signals and mechanical rupture. Regardless of the spatiotemporal scale, rock rupturing generally progresses through four distinct phases: crack initiation from intact rock (or complete closure of preexisting cracks), crack opening, crack dilation, and crack propagation (states I–IV, Fig. 3*A*). Within this framework, we propose that in state I, although *S* exists and the conditions for nuclide genesis are satisfied, the crack remains unopened, and the exposed surface area *S*_e_ for nuclide release is zero. As a result, no nuclide signal can emanate. Subsequently, the crack opens and *S*_e_ > 0, triggering a nuclide signal burst. Thus, states I and II represent a critical transition for the onset of nuclide signal release. In the following discussion, we adopt *S*_e_ instead of *S*, as it more effectively quantifies the control of crack area over nuclide signals. Furthermore, crack dilation is characterized by an increase in *b* while *S*_e_ remains relatively constant. Conversely, crack propagation involves an abrupt increase in *S*_e_ due to new crack growth (Fig. 3*A*), which significantly outpaces its volumetric enlargement (i.e., *b* remains relatively constant) (33–35). In this context, we develop a physical model to describe signal evolution across these complete rupturing states. During the transition from states I to II, nuclide signals are released from nascent cracks primarily via *α*-recoil from crack surfaces, a process quantified by the recoil production rate (*P*_r_). The influence of rock lithology on nuclide signals can be primarily quantified by *P*_r_, since its effect is mainly reflected in the varying parent nuclide contents of different mineral compositions. During crack dilation (states II to III), the enlargement of *b* induces signal attenuation approximated with the cubic law of fluid flow (29, 36), as enhanced flushing by the fluid carrier promotes nuclide removal. In contrast, crack propagation (states III to IV) triggers sudden nuclide signal spikes, driven by the abrupt expansion of *S*_e_ and the subsequent enhancement of *P*_r_ (2, 21, 30).

Establishing constitutive relations rely on quantitative relationships to relate nuclide signals to rupture states. We achieve this by analytically computing nuclide concentrations and fluxes (i.e., the nuclide signals) which accompany rock rupture. The analytical framework employs a one-dimensional (1D) model along the longitudinal signal transmission axis that coincides with the principal rupture direction. In the 1D framework, the cumulative signal transmissions during stochastic, disordered rock rupture events are homogenized through equivalent physical representations (Fig. 3*A*). Specifically, the increase in crack intensity (quantified by *S*_e_) is equivalent to an increase in transverse uniform recoil input (quantified by *P*_r_), while the enhanced nuclide flushing from expanded crack volume (quantified by *b*) corresponds to a rise in the longitudinal equivalent *ϖ* (illustration of this equivalence is provided in *SI Appendix*, Fig. S3). ^222^Rn is selected as the representative nuclide, as it encapsulates the key processes universally inclusive to other nuclides, including alpha recoil, decay (quantified by decay rate *λ*), diffusion, and advection, and is easiest to measure. ^222^Rn is also the most commonly documented radionuclide in field settings (20, 24, 37–39).

The derived analytical solutions include the temporal evolution of *A*, as well as the analytical expressions for *A*_eq_ and *A*_max_ (details of these expressions are provided in *Section* 2 of M&M). Building upon these solutions, we quantitatively characterize the evolutionary pathway of nuclide signals through all the rupturing states: from initial signal release at crack opening, through signal attenuation during crack dilation, to subsequent signal surge upon crack propagation (Fig. 3*B*). The analytical model provides a quantitative description of the mechanisms depicted in Fig. 3*A*, and establish a theoretical basis for deriving constitutive relationships. On this basis, we further develop two constitutive equations (Eqs. **10** and **13**, *Section 3* of M&M) that describe how *A*_eq_ and *A*_max_, which serve as practical metrics for forecasting scenarios, respond to changes in *b* (representing crack dilation) and *S*_e_ (representing crack propagation), respectively. These two constitutive relations are validated within a generalized framework through high-resolution pore-scale numerical simulations (with details of the simulation method provided in *Section 4* of M&M, and validations provided in *Section 5* of M&M). The validation encompasses a wide range of conditions, including representative naturally occurring nuclide pairs (^226^Ra~^222^Rn and ^228^Ra~^224^Ra) with comprehensive physicochemical representation (*SI Appendix*, Fig. S4), varying rupture crack properties (from smooth to rough surfaces), and diverse flow regimes (from Poiseuille flow to heterogeneous velocity fields). The simulations confirm the general applicability of the constitutive model.

During the construction of the physical model and constitutive relationship, we divide the rock rupture process into four stages, namely states I-IV. This division is designed to match the key rupture characteristics that control the nuclide signals. In terms of their sequential occurrence, states I-IV do not need to follow a strict unidirectional progression, and some states may often be coupled and difficult to isolate completely, such as crack dilation and propagation. Therefore, in the practical application of the established constitutive relations, the definition of states may involve selecting a dominant state, or a trade-off among these states. This trade-off is guided by the criterion of optimally characterizing the evolution of the nuclide signal under the premise of satisfying physical mechanisms.

### Rock Rupture Diagnostic Theory.

By manipulating the two constitutive relations (Eqs. **10** and **13**, M&M) we can derive their inverse functions to relate *A*_eq_ and *A*_max_ to changes in *b* and variations in *S*_e_,

[1a] $$b=f^{-1}\left(A_{\mathrm{eq}}\right)=\frac{q}{\lambda L}10^{-\left(A_{\text{eq}}^{*}/\kappa +Zh_{0}\right)},$$

[1b] $$S_{\text{e}}=h^{-1}\left(A_{\max}\right)=\frac{1}{\Gamma }\left(A_{\max}-A_{0}\right),$$

where $A_{\mathrm{eq}}^{*}$ is normalized equilibrium signal defined as ln(*A*_max_/*A*_eq_−1), *κ* and *Zh*_0_ are dimensionless parameters, *A*_0_ (unit: dpm⋅L^−1^ when dpm is disintegrations per minute) incorporates contributions from additional reactions, and Γ (= 0.25*r$A_{U238}^{r}$ρ*_s_Λ/*V*_REV_, unit: dpm⋅L^−1^⋅m^−2^) represents the areal nuclide production rate due to *α*-recoil, *r* is *α*-recoil range, *ρ*_s_ is rock density, and $A_{U238}^{r}$ is activity concentration inside mineral lattice in fresh rocks. Detailed derivations of Eqs. **1a** and **1b** are provided in *Section 3* of M&M.

In the diagnostic theory (Eqs. **1a** and **1b**), *A*_max_ represents the equilibrium nuclide signal under crack opening or no-flow conditions (*ϖ* → 0, corresponding to state II in Fig. 3*A*). A sudden increase in *A*_max_ suggests that the rupture area is expanding due to crack propagation (Fig. 3*B*). Therefore, by tracking how *A*_max_ changes over time, we can estimate variations in *S*_e_. On the other hand, normalized signal $A_{\mathrm{eq}}^{*}$ reflects how much the current dilation state (state III) deviates from the initial state (state II), capturing information about the rupture aperture. A decline in $A_{\mathrm{eq}}^{*}$ (i.e., *A*_eq_) indicates crack dilation and the resulting enlargement of the aperture, which pulls *A*_eq_ away from *A*_max_ (Fig. 3*B*). Monitoring the evolution of *A*_eq_ thus allows us to track changes in *b*. By combining these two signals, fluctuations in *A*_eq_ (caused by crack dilation) and spikes in *A*_max_ (caused by crack propagation), Eqs. **1a** and **1b** enables the diagnosis of the evolution of rock rupture.

Previous studies have provided pioneering contributions to modeling nuclide release during rock deformation (2, 5, 40). As a representative framework, the dynamic dual-permeability model captures deformation-induced variations in hydraulic properties by incorporating time-dependent permeability and porosity, thereby allowing the simulation of nuclide signal evolution (5). These models focus primarily on nuclide variations driven by rock deformation, which aligns with one component of the constitutive relation developed in this study (Eq. **1b**). However, most existing models are formulated at the macroscopic Darcy scale and remain limited to governing-equation-level descriptions, and do not provide an inverse framework for directly inferring rupture characteristics. Building on these model foundations, our study, proceeding from the pore scale and analytical support, further incorporates the physical process by which rock rupture increases crack surface area. This enhancement allows more target nuclides, emanating from the decay of parent nuclides within rock mineral grains, to be released into the crack domain, thus elevating the nuclide genesis rate. Accordingly, the key distinction of our model lies in the introduction of a nuclide flux into the pore space, represented by the recoil term *P*_r_ that is linked to *S*_e_. This physical formulation enables the incorporation of crack surface area, a critical descriptor of rock ruptures, into the nuclide evolution equation.

### Diagnosis of Rock Progressive Failure in Laboratory Settings.

Our diagnostic theory is first applied to a published month-long laboratory experiment (2), with the observed signals shown in Fig. 2*A*. The radon signal evolution exhibits distinct evolutionary phases: stepwise intensification-stabilization cycles at the beginning (days 5-21), followed by abrupt intensification (days 21-25), and stepwise decline-stabilization at the end (days 25-30) (Fig. 2*A*). Based on our diagnostic theory, we infer that these evolutionary signals indicate the crack network of the rock sample experienced crack closure (states III → II), crack propagation (states III → IV), and crack dilation (states II → III). This inferred evolutionary pathway coincides with the variations of equivalent *b* derived from the measured permeability (Fig. 2*A*).

**Fig. 4.**
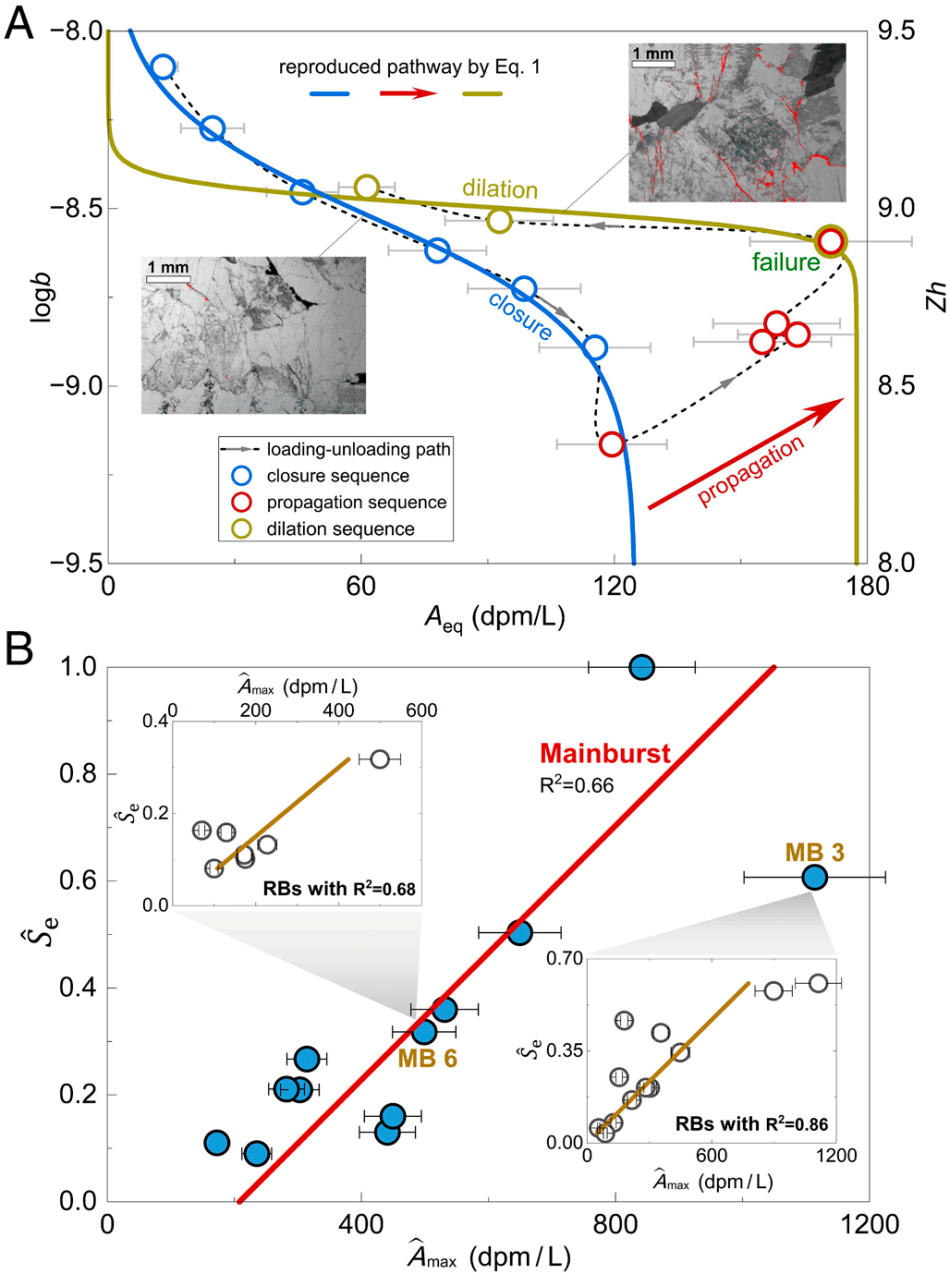
Signatures of rock failure at laboratory and field scales. (*A*) Rock property evolution in terms of rupture aperture (quantified by log*b*) and *A*_eq_. Experimental results are derived from the observed radon signal time series shown in Fig. 2*A*. *Zh* = log (*ϖ*/*λ*) = log(*q*/*λbL*) number is a mapping function of *b*. The error bars in signals are the fluctuation range of the equilibrium signals. *A*_eq_ exhibits phased evolutionary characteristics, responding to three distinct rupturing state transitions: crack closure, crack propagation, and crack dilation (sequentially illustrated in Fig. 3*A*). Both crack closure and dilation sequences can be well replicated by Eq. **1a** with R^2^ = 0.99 and 0.64, respectively (fitted parameters listed in *SI Appendix, Text S6*). A prominent increase in *S*_e_ after the sample failure is evidenced by optical photographs (*Insets* where red regions indicate crack surfaces). Details of the laboratory experiment and data analysis are provided in *SI Appendix*, Table S5 and *Text S4*. (*B*) Rock property evolution in a field setting during a sequence of rock rupturing events, in terms of apparent rupture area ${\hat{S}}_{e}$ and transient pulse ${\hat{A}}_{max}$. Field-scale results are derived from observed radon signal time series shown in Fig. 2*B*. Both main burst (MB) and residual burst (RB) events can be well replicated by Eq. **1b** with R^2^ = 0.66 ~ 0.86 (fitted parameters listed in *SI Appendix, Text S8*). The error bars in *B* are evaluated based on the 10% temporal uncertainty of the radon monitorings device BARASOL (20, 37). Details of the field observations and data analysis are provided in *SI Appendix*, Table S7 and *Text S5*.

Based on the above diagnostics, we further demonstrate how our diagnostic theory reconstructs the complete processes of rock rupture using nuclide signals. We extract the averaged equilibrium radon signals (*A*_eq_, Fig. 2*A*) under various stressed conditions and plot them against *b* in Fig. 4*A*. A logarithmic scale is used for *b* to improve visual clarity. We find that, during crack closure (states III → II), *A*_eq_ rises nearly by an order of magnitude (gradually increasing from 13.6 to 125.0 dpm/L), signifying a decrease of *b* from 8.4 × 10^−3^ to 7.3 × 10^−4^ mm. Eq. **1a** successfully tracks this crack closure sequence, with R^2^ = 0.995 and *P* < 0.0001 (Fig. 4*A*; fitted parameters provided in *SI Appendix*, Table S5). Subsequently, when crack propagation turns to sample failure (states III → IV), radon signals surge sharply (from 125 to nearly 180 dpm/L). Optical photographs (ref. 2; *Inset* of Fig. 4*A*) show that the crack network of the sample transitioned from a microcrack-dominated (prefailure) to a macrocrack-dominated (postfailure) regime. Following the failure, *A*_eq_ reduces by half (from nearly 180 to 97 dpm/L), signifying an increase of *b* from 2.7 × 10^−3^ to 3.9 × 10^−3^ mm. Eq. **1a** also effectively replicates this crack dilation sequence, with R^2^ = 0.814 and *P* < 0.05 (Fig. 4*A*; fitted parameters provided in *SI Appendix*, Table S5). Moreover, comparison of the fitted *A*_max_ values reveals that the postfailure *A*_max_ is approximately 42% higher than the prefailure value, signifying a significant increase in *S*_e_. This distinct *S*_e_ change is consistent with the crack network evolution visually documented in images of the sample (*Inset* of Fig. 4*A*). These experimental results support our theory’s ability to track crack dilation, closure, and crack propagation at a representative elementary volume scale.

### Interpretation of Bedrock Deformation at the Field Scale.

We next apply our theory to interpret published monitoring data from bedrock hillslope deformation triggered by reservoir impoundment (20). Radon signals exhibit periodic burst patterns (i.e., transient pulse), where each main radon burst is consistently followed by a series of moderate or minor or residual bursts (Fig. 2*B*). According to our diagnostic theory, these radon burst patterns could be attributed primarily to an increase in *S*_e_ of the crack network, which leads to a rapid rise in the nuclide genesis rate. This increase in *S*_e_ likely arises from two main factors. First, water impoundment elevates pore pressure, thereby enhancing the connectivity of preexisting cracks. This mechanism has also been thoroughly discussed and analyzed in ref. 20 as an important trigger for radon bursts.

Here, based on field-observed transient nonlinear elastic responses from tiltmeter measurements under impoundment loading during the radon burst (figure 4 in ref. 20), we infer an additional source for radon bursts: a steep increase in *S*_e_ caused by a sequence of crack propagation events. We draw this inference because nonlinear elastic or anelastic deformation indicates that the rock has already entered the stable crack growth stage (33, 41). Furthermore, numerous case studies have demonstrated that large-scale reservoir impoundment induces rock damage or even instability (42, 43), and these processes are accompanied by extensive crack propagation. Considering these facts together, and given the nearly 40-fold sudden increase in radon signals, we propose that a sequence of crack propagation events occurred at the Roselend site, and contributed to the observed radon burst. Our analysis further suggests that the main burst and its residual evolution may represent either distinct phases of primary and secondary crack propagation (and/or the interconnection of preexisting cracks) (44–46), or alternatively a transition in crack propagation dynamics. Such a transition can result from either primary forcing (e.g., rapid reservoir water-level fluctuations) or from secondary environmental triggers, such as changes in temperature and humidity, or rainfall events, as exemplified by ref. 20.

Our theory enables quantitative tracking of field-scale deformation events within a diagnostic framework. We extract transient pulse ${\hat{A}}_{max}$ (the approximation of maximum radon signal) from each radon burst event, based on the premise that monitoring data primarily reflect bedrock surface emanation dominated by crack-controlled radon diffusion, with minimal influence from advective transport (*SI Appendix, Text S5*). Changes in *S*_e_ of the bedrock crack network are manifested by variations in macroscopic tilt, highly correlated with the acceleration rate of water level fluctuations (20). Therefore, in the light of ref. 20, we reconstruct the dynamics of the apparent exposed rupture area (${\hat{S}}_{e}$) at the observation site, using the second time-derivative of the recorded reservoir water level fluctuations (*SI Appendix, Text S5*). Analysis of 12 typical main burst events (green circles in Fig. 2*B*) reveals a correlation between ${\hat{A}}_{max}$ and ${\hat{S}}_{e}$ extracted from the observations. When ${\hat{A}}_{max}$ varies by one order of magnitude (increasing from 172.5 to 1,113.8 dpm/L), ${\hat{S}}_{e}$ exhibits synchronous order-of-magnitude growth, signifying a series of primary crack propagation and/or connection events. Additionally, this significant correlation is also observed in residual radon burst events (circle points in Fig. 4*B*, *Insets*), signifying a series of secondary crack propagation and/or connection events. By reproducing the monitored ${\hat{A}}_{max}$ ~ ${\hat{S}}_{e}$ data series via Eq. **1b**, our theory diagnoses all primary crack propagation and/or connection events (main burst events *n* = 12, Fig. 4*B*), with R^2^ = 0.66 and *P* < 0.005. Furthermore, Eq. **1b** in the theory also effectively tracks subsequent secondary crack propagation and/or connection sequences following two main radon bursts (residual burst events *n* = 12 and 7 with R^2^ = 0.86 and 0.96, respectively, *P* < 0.0001 for both). These diagnostic results demonstrate the application potential of our theory for interpreting field-scale rock deformation and failure.

## Discussion

Rock rupture, particularly brittle failure, is characterized by minimal prefailing strain, such that many geohazards are both difficult to detect and have an apparently sudden onset. These features pose significant challenges for detection and prediction via conventional methodologies. However, naturally occurring nuclide signals released during rupture are characterized by high sensitivity and exceptional detectability with modern instrumentation, and hence offer a promising way for diagnosing failure events.

In this study, we establish and apply a paradigm unit for the release and evolution of nuclide signals during rock rupture (Fig. 1). By decomposing observed nuclide time series into a set of paradigm units (Fig. 2), we successfully extract geomechanical information. Each decomposed paradigm unit represents a discrete microrupturing event occurring at a specific time and scale. Systematically analyzing these units allows us to reconstruct the complete microrupturing sequence, including event density and timing, within critical systems such as seismic faults and unstable slopes. Our proposed decomposition and reconstruction methods offer opportunities for future studies that could integrate multidisciplinary constraints, such as acoustic emission monitoring (44, 47), to calibrate and validate these signal paradigm units. At the core of quantitatively deciphering rock rupture is the interpretation of two signal characteristics: *A*_max_ and *A*_eq_. Our findings indicate that *A*_max_ effectively captures abrupt expansions in surface area, while *A*_eq_ reflects variations in crack aperture. Leveraging these findings, we develop a quantitative theory for diagnosing rock rupture (Eqs. **1a** and **1b**) through a combination of theoretical models, numerical simulation, and multiscale applications.

Our framework tracks *S*_e_ and *b*, which serve as structural indicators of rock damage and geological stability (33, 48, 49). Meanwhile, *S*_e_ and *b* can also be upscaled to their influence on macroscopic hydraulic conductivity and seismic velocity of rock masses. Specifically, the expansion of *b* during rupturing reflects crack volume growth and is linked to permeability enhancement (29, 36), which can trigger cascading earthquakes (50, 51). Given that permeability often scales linearly with peak ground velocity (a proxy for dynamic strain) (52), our diagnostic model for *b* provides a benchmark for tracking the dynamic strain fields of cascading seismic events. Similarly, an increase in *S*_e_ signifies the intensification of crack networks and directly correlates with earthquake dynamics (53, 54). This relationship is exemplified by the proportionality between *S*_e_^3/2^ and seismic moment (48); consequently, our diagnostic model for *S*_e_ facilitates the estimation of fault slip dynamics.

Given the strong geometric heterogeneity inherent to fractured media, scale effects remain a ubiquitous challenge in studies of subsurface mechanics, flow, and transport processes (55–59). Consequently, this issue provides a challenge to map observational data onto theoretical models. The constitutive Eqs. **1a** and **1b** established in this study are rooted in both single-crack models (encompassing ideal parallel plates and real rough cracks) and upscaled 1D models for crack networks at REV scale. Like the cubic law in classical single-crack flow theory (29, 36), Eqs. **1a** and **1b** serves as a fundamental theory for investigating crack network-related problems. Therefore, extending Eqs. **1a** and **1b** to crack network systems can draw extensively on the well-established upscaling methodologies developed for the cubic law (57, 60–62). Furthermore, the theoretical formulations proposed in this study incorporate physical quantities that hold potential for evaluating scale effects. Specifically, the key dimensionless number *Zh* in Eq. **1a** explicitly includes the length *L*, laying a foundational basis for analyzing scale effects induced by crack length variations. The introduction of the representative elementary volume *V*_REV_ in Eq. **1b** provides another avenue for future scale effect analysis, particularly through the incorporation of fractal and statistical distribution models for crack networks (55). Although the core physical variables in this study (crack aperture and crack area) are defined at the single-crack scale, they can be directly linked to the bulk permeability and crack intensity *ρ*_3D_ (defined as the total crack area per unit volume) of the entire crack network, respectively (57, 62). While the theoretical model constructed herein demonstrates potential for cross-scale extension, systematic research is still needed to further test and implement such extensions.

There are additional complexities at the field scale that are not captured in our physical model (Eqs. **14a** and **14b** and *SI Appendix*, Fig. S4). For instance, fluid remobilization and mixing between distinct reservoirs or aquifers may affect nuclide signals, particularly for deep fluids, thermal waters, or brines (5, 7, 63). Owing to their high salinity or temperature, such fluids generally feature enhanced nuclide desorption and mobility, resulting in elevated nuclide signals (63–65). When rock rupture connects to these deep fluid pathways, fluid remobilization and mixing can significantly amplify the observed nuclide signals. In such scenarios, our physical model needs to be extended by incorporating additional source-sink terms and initial and boundary conditions into the governing equation (Eqs. **14a** and **14b**), which can be further integrated into the constitutive relations (Eqs. **1a** and **1b**).

From the perspective of rock rupture diagnosis, there exists another highly important and practically significant issue, namely the timeliness of diagnosis, which determines the time window for the early warning of geohazards associated with rock ruptures. A case in point is the M 7.2 Kobe Earthquake that occurred in 1995, where a more than 10-fold increase in radon signals was observed as early as 9 d prior to the earthquake (13). Our study also involves the quantification of the time scales of signal transmission, such as the decay timescale between *A*_max_ and *A*_eq_ in the paradigm unit (which can be quantitatively analyzed using fitting parameters in *SI Appendix*, Table S1), and the equilibrium time (*t*_c_) of the breakthrough curve in signal transmission simulations (which can be quantitatively analyzed based on *SI Appendix*, Figs. S9 and S10).

Although practical challenges remain for rock rupture forecast, the nuclide signal decomposition and rupturing models proposed in this study offer theoretical support for rock rupture forecasting from two perspectives: forecast validity and forecast timeliness. We illustrate this using a flowchart framework for rock rupture forecasting. First, a representative and comprehensive real-time nuclide signal monitoring system should be established to obtain long-term nuclide time series. The proposed nuclide signal decomposition method is then applied to extract the characteristic signals *A*_max_ and *A*_eq_. Using the constitutive Eqs. **1a** and **1b**, the rupture state in terms of aperture and area can be quantified. These two physical quantities serve as key indicators for assessing rock damage and its evolution, providing a framework to interpret changes. Meanwhile, this study provides a quantitative approach for evaluating forecast timeliness. As *A*_eq_ is used to track rupture aperture, the equilibrium of the nuclide signal (quantified by *t*_c_) constitutes an important prerequisite for theoretical validity. Our preliminary analysis shows that *t*_c_ can be well represented by the dimensionless number *Zh* across various nuclides (*SI Appendix*, Fig. S13). Since *Zh* can be derived from *A*_eq_ (Eq. **1a**), *t*_c_ can be further estimated from *A*_eq_, which provides an important basis for evaluating forecast timeliness.

Despite its potential forecasting implications, this study is still positioned to probe rock rupture rather than provide true forecasting. Rock rupture forecast, especially at the field scale, remains a formidable challenge where there might not be a single indicator for precise forecasting. The most effective forecast will only be achieved through the integrated analysis and mutual corroboration of data from multiple monitoring data streams. Therefore, this study does not aim to replace existing rock deformation and failure monitoring techniques, such as acoustic emission or stress and strain measurements. Instead, we seek an approach that adds additional insights in a monitoring framework.

The proposed signal decomposition and diagnostic theory connect the mesoscopic interpretation of microrupturing with the macroscopic prediction of rock deformation and failure. Together, these advances establish a diagnostic framework that can help remove some of the critical barriers along the pathway of *nuclide signal monitoring* → *crack growth identification* → *geohazard diagnosis and forecast*. With further multidisciplinary integration of rock mechanics, seismology, and engineering geology, there may be opportunities to ultimately achieve catastrophic state assessment and early warning for rupture-induced geohazards. The model highlights the value in collecting nuclide data in the lab and field.

## Materials and Methods (M&M)

### Section 1. Observed Nuclide Signal Time Series and Its Decomposition.

To assess our modeling framework, we analyzed two representative radon signal time series spanning laboratory and field scales. The first is a month-long laboratory experiment conducted by Girault et al. (2), who loaded an intact granite cylinder, treated as representative elementary volume (REV), until failure under dry, upper-crustal conditions. Their study continuously monitored permeability evolution and radon emissions throughout the loading-unloading path (details of the laboratory experiments provided in *SI Appendix, Text S5*). The second is a three-year monitoring campaign by Trique et al. (20) on a bedrock hillslope adjacent to a reservoir. Given that periodic 75-m water-level fluctuations could induce significant bedrock deformation, radon dynamics were tracked alongside inclinometer measurements to capture potential failure events (details of the field scale observations provided in *SI Appendix, Text S6*).

For decomposing the observed radon time series, we define a piecewise asymmetric logistic function, *A_i_*(*t*) (Eq. **2a**), as the basic function of the paradigm unit, for representing single microrupturing event (Fig. 1*B*). This function is governed by two sets of parameters: signal intensity parameters (*A*_max,_*_i_* and *A*_eq,_*_i_*) and temporal evolution parameters (*χ_i_*, *γ_i_*, *τ*_0,_*_i_*, *τ*_peak,_*_i_*). Note that the index *i* in the variables refers to the *i*-th microrupturing event. The cumulative signal *A*(*t*) is modeled as the temporal superposition of multiple basic functions:

[2a] $$\begin{array}{c} A_{i}(t)=\left\{\begin{array}{c} \frac{A_{\text{max},i}}{1+e^{\left[-\chi_{i}\left(t-\tau_{0,i}\right)\right]}}\\ \tau_{\text{start},i}\leq t\leq \tau_{\text{peak},i}\\ A_{\mathrm{eq},i}+\left(A_{\text{max}\text{,}i}-A_{\mathrm{eq}\text{,}i}\right){\text{e}}^{\left[-\gamma_{i}\left(t-\tau_{\text{peak},i}\right)\right]}\\ \tau_{\text{peak},i}<t\leq \tau_{\text{end},i} \end{array}\right., \end{array}$$

[2b] $$A(t)=\sum_{i=1}^{N}w_{i}A_{i}\left(t\right).$$

The choice of this function carries a clear physical meaning. Specifically, the function used to characterize the paradigm unit should be able to capture two key features: the nuclide signal pulse caused by the increase in genesis rate (controlled by *S*_e_), and the attenuation of the nuclide signal caused by the increase in flushing rate (controlled by *b*). These characteristics are derived from and motivated by the observed radon signals shown in Fig. 2, based on both laboratory and field observations. Under these physical constraints, any characterizing function must be constrained by the two key signal features: the peak amplitude (*A*_max_) and the equilibrium amplitude (*A*_eq_). While we acknowledge that Eqs. **2a** and **2b** may not be the only mathematical form capable of representing these coupled mechanisms, this nonuniqueness does not affect the validity of our subsequent analysis. The primary purpose of Eqs. **2a** and **2b** is to establish a bridge between characteristic nuclide signals and the underlying rupture structural parameters. It is expected to provide a mechanistic basis for formulating the constitutive relations between nuclide signals and rupture in the subsequent analysis.

To prevent the unrealistic accumulation of steady-state signals over long durations, we define a finite life-cycle for each microrupturing event, bounded by time $\tau_{\mathrm{start}}^{i}$ and $\tau_{\mathrm{end}}^{i}$. Physically, this assumes that the contribution of a single microrupture to the macroscopic signal is intermittent and limited to a specific duration, after which it no longer impacts the cumulative baseline. The number of basic functions (*N*) is initialized based on the frequency of external perturbations (*N*_0_). In the laboratory test, *N*_0_ = 13 (comprising one gas injection and 12 loading-unloading cycles, green circles in Fig. 2*A*), while in the field observation, *N*_0_ = 12 (corresponding to 12 main bursts, green circles in Fig. 2*B*). Recognizing that each perturbation may trigger multiple microrupturing events, we expand the number of events exponentially: *N* = *N*_0_ × 2*^m^*. These *N* events are assumed to be distributed evenly across the total observation time *T*.

For each basic function, the weight *w_i_* of *A_i_*(*t*) is determined by the local macroscopic signal intensity at *τ*_peak,_*_i_*, such that *w_i_* = *A*(*τ*_peak,_*_i_*)/max(*A*) × (*δ*/*N*), where *δ*/*N* is a correction coefficient. The steady-state value *A*_eq,_*_i_* is treated as a reduction of *A*_max,_*_i_*, defined as *A*_eq,_*_i_* = *φA*_max,_*_i_*, where *φ* fluctuates within a physically plausible range. By iteratively increasing the density of basic functions (adjusting *m*) and optimizing the parameter set (*χ_i_*, *γ_i_*, *τ*_0,_*_i_*, *τ*_peak,_*_i_*, *τ*_start,_*_i_*, *τ*_end,_*_i_*, *δ*, *φ*), we minimize the root-mean-square error until it stabilizes (*SI Appendix*, Figs. S1*A* and S2*A*). This allows us to determine the minimum number of paradigm units required to accurately reconstruct the time series. Applying this method to the laboratory rock deformation and failure, we decompose the signal into 832 paradigm units, achieving a reconstruction accuracy of R^2^ = 0.96 (Fig. 2*A* and *SI Appendix*, Fig. S1*B*). For the hillslope deformation, the signal was decomposed into 6,144 paradigm units with a reconstruction accuracy of R^2^ = 0.87 (Fig. 2*B* and *SI Appendix*, Fig. S2*B*). Estimated parameters of the signal decomposition are provided in *SI Appendix, Text S1*.

### Section 2. Analytical Solutions for Radon Signals in Response to Rock Rupture.

Our analytical solution is derived from a one-dimensional (1D) radon (^222^Rn) transmission model, designed to quantify how nuclide concentrations (i.e., signal intensity) change at the flow outlet during rock rupture. Radon is employed here because it involves representative physicochemical processes (advection, diffusion, recoil, and decay) that are universally applicable to all radionuclides, while remaining amenable to closed-form mathematical solutions. The signal transmission direction coincides with the main rupture direction. Our 1D model adopts this as the principal axis (with a length *L*), serving as a simplified representation of cumulative signal transmissions during stochastic and disordered rupture events (Fig. 3*A* and *SI Appendix*, Fig. S3).

Rock rupture produces two primary impacts on signal evolution: they increase crack surface area, enhancing nuclide genesis along the transmission path; and they enlarge crack volume, intensifying convective renewal and accelerating nuclide flushing. In our 1D framework, these mechanisms are captured through variations of two parameters: the increase in crack area (quantified by *S*_e_) corresponding to the enhanced transverse uniform recoil (*P*_r_) input, and the enlargement of crack volume (quantified by *b*) linked to an increased longitudinal flushing rate (*ϖ* = *v*/*L*, *v* denotes the flow velocity) (*SI Appendix*, Fig. S3). In this context, the governing equation for 1D radon transmission during rock rupture is

[3] $$\frac{\partial C}{\partial t}=D\frac{\partial^{2}C}{\partial x^{2}}-\varpi L\frac{\partial C}{\partial x}-\lambda C+P_{r},$$

where *C* = *A*/*λ* [atom⋅L^−3^] denotes the concentration, *A* [atom⋅L^−3^⋅T^−1^] denotes the radon activity, *D* [L^2^⋅T^−1^] denotes the diffusion coeffi.cient of radon, *λ* [T^−1^] denotes the decay rate. The model employs a zero-^222^Rn concentration boundary at the inlet, a free outflow boundary at the outlet, as well as zero-concentration initial condition. The governing Eq. **3** and initial and boundary conditions describe radon evolution in a newly formed crack without external sources.

The analytical solution of Eq. **3** can be obtained using the method of separation of variables (66), with derivation details provided in *SI Appendix, Text S1*. Final form of the solution is

[4a] $$C\left(x,t\right)=\frac{P_{r}}{\lambda }g_{1}\left(x,t;D,\lambda ,\varpi ,L\right),$$

[4b] $$\begin{array}{c} g_{1}=e^{\varpi Lx/2D}\cdot \sum_{n=1}^{\infty }\frac{1}{\int_{0}^{L}{\text{sin}}^{2}\left(xk_{n}\right)\text{d}x}\cdot \frac{k_{n}}{{\left(\varpi L/2D\right)}^{2}+k_{n}^{2}}\\ \cdot \frac{\lambda }{Dk_{n}^{2}+{\left(\varpi L\right)}^{2}/4D+\lambda }\cdot \left(1-e^{-\left(Dk_{n}^{2}+\varpi^{2}L^{2}/4D+\lambda \right)t}\right)\text{sin}\left(xk_{n}\right), \end{array}$$

where the function *g*_1_ [-] is an infinite series, and *k_n_* [L^–1^] denotes the eigenvalues of Eq. **3** and is given by Eq. S14 in *SI Appendix, Text S1*.

By substituting *x* = *L* into Eq. **4**, the radon signal *A* *(= *λC**) at the outlet (*x* = *L*) can be expressed as

[5] $$A=\lambda P_{r}g_{2}\left(t;D,\lambda ,\varpi ,L\right).$$

This solution quantifies the breakthrough curves (BTCs) at the outlet in response to changes in *ϖ* and *P*_r._

As time *t* approaches infinity, the expression for the equilibrium signal *A*_eq_ at the crack outlet can be obtained from Eq. **5**

[6] $$A_{\text{eq}}=\lambda P_{r}g_{3}\left(D,\lambda ,\varpi ,L\right).$$

According to Eq. **6**, *A*_eq_ increases as *ϖ* decreases, reaching a maximum value (*A*_max_) when the flow vanishes (*ϖ* ≈ 0)

[7] $$A_{\text{max}}=\lambda P_{r}g_{4}\left(D,\lambda ,L\right).$$

In a rock rupturing context, *A*_max_ corresponds to *A*_eq_ when the crack opens from complete closure or initiation and advection is negligible (state II illustrated in Fig. 3*A*).

Eqs. **4**–**7** provide general expressions for radon accumulation and concentration evolution during rock rupture, along with characteristic signals (*A*_eq_ and *A*_max_) at the signal exit. The expressions for functions *g*_2_ to *g*_4_ in Eqs. **5**–**7** correspond to specific substitutions of variables into Eq. **4b**. Their detailed mathematical forms are provided in *SI Appendix, Text S1*.

### Section 3. Constitutive Relations between Nuclide Signals and Ruptures.

The constitutive relations were developed based on the analytical solutions for nuclide signals released from rock rupture (*Section 2*). Specifically, to examine how *b* (quantified by *ϖ*) affects *A*_eq_, we use Eq. **6** to compute *A*_eq_ across various *ϖ* values. The results reveal an approximately linear relationship between the normalized equilibrium signal $A_{\mathrm{eq}}^{*}$ = ln(*A*_max_/*A*_eq_−1) and a defined dimensionless number *Zh* (*SI Appendix*, Fig. S6), yielding

[8] $$A_{\text{eq}}^{*}=\kappa \left(Zh-Zh_{0}\right),$$

where $A_{\mathrm{eq}}^{*}$ represents the deviation from the initial state (stage II in Fig. 3*A*) to the dilation state (stage III in Fig. 3*A*). *Zh* compares the flushing rate (*ϖ*) to the decay rate (*λ*), defined as

[9] $$Zh=\text{log}\left(\frac{\varpi }{\lambda }\right)=\text{log}\left(\frac{q}{\lambda bL}\right).$$

*Zh* serves as a mapping function of *b* or a normalized value of *ϖ*, and reflects the influence of aperture variation on the nuclide signal during crack dilation. The log transformation in the definition of *Zh* facilitates eliminating decay background effects and normalizes the wide span of half-lives into a comparable scale.

By rearranging Eq. **8**, we can derive the constitutive equation between *A*_eq_ and *b*:

[10] $$A_{\mathrm{eq}}=f\left(b\right)=\frac{A_{\max}}{1+e^{\kappa \left(Zh-Zh_{0}\right)}},$$

where the slope *κ* quantifies the rate at which *A*_eq_ deviates from *A*_max_ as the crack dilates, and *Zh*_0_ is the characteristic point where *A*_eq_ equals half of *A*_max_. The inverse form of this constitutive equation yields the diagnostic formula for *b* (Eq. **1a**).

Similarly, Eq. **7** theoretically supports linking *A*_max_ to *S*_e_ (quantified by *P*_r_). Eq. **7** shows that *A*_max_ is proportional to *P*_r_ with a dimensionless slope Λ = *λg*_4_

[11] $$A_{\max}=\Lambda P_{\text{r}}+A_{0},$$

where the slope Λ integrates the effects of *L*, *D,* and *λ*, characterizing the signal sensitivity to crack propagation; *A*_0_ accounts for other reaction contributions, zero in this analysis but potentially nonzero in other contexts (processes 2–4 and 6–8 illustrated in Fig. 1*B*). Λ follows a logistic curve with *L* and stabilizes near 1 for *L* > 0.1 m (*SI Appendix*, Fig. S7), the value used in this study.

Therefore, during crack propagation, *A*_max_ is mainly controlled by *P*_r_, which in turn is positively correlated with *S*_e_ (2). This correlation between *P*_r_ and *S*_e_ can be captured using the following relationship (21, 30, 67)

[12] $$S_{e\text{sa}}=\frac{P_{\text{r}}}{0.25rA_{\text{U}238}^{\text{r}}\rho_{\text{s}}},$$

where *S*_esa_ is effective specific surface area, *r* is *α*-recoil range (∼0.05 µm), *ρ*_s_ is rock density, and $A_{U238}^{r}$ is activity concentration inside mineral lattice in fresh rocks. Introducing a representative elementary volume (*V*_REV_), we write *S*_e_ = *S*_esa_⋅*V*_REV_, leading to

[13] $$A_{\max}=h\left(S_{\text{e}}\right)=\Gamma S_{\text{e}}+A_{0},$$

where Γ = $0.25rA_{\text{U}238}^{\text{r}}\rho_{\text{s}}$Λ/*V*_REV_ (dpm⋅L^−1^⋅m^−2^) represents the nuclide production rate per unit area by *α*-recoil. Eq. **13** establishes a constitutive relation between *A*_max_ and *S*_e_. The inverse form of this constitutive equation yields the diagnostic formula for *S*_e_ (Eq. **1b**).

It is important to emphasize that Eq. **13** introduces a key physical concept: *V*_REV_ (illustrated by the pink dashed box in Fig. 1*A*). *V*_REV_ refers to the volume of the domain relevant to the data being interpreted. At the laboratory sample scale, *V*_REV_ corresponds to the specimen volume, such as the volume of the cylindrical granite specimen involved (Fig. 2*A*). At the field scale, such as for earthquakes or landslides, *V*_REV_ represents the volume of the seismic fault zone and surrounding fractured rock, or of the slip zone and its adjacent influenced region. Regardless of scale, *V*_REV_ is determined by both the signal transmission path length *L* and the cross-sectional extent that affects signal transmission. In a field setting, *V*_REV_ and *L* are not known a priori, while certain estimations would be made by means of borehole testing and geophysical exploration methods (47, 68, 69).

### Section 4. Pore-Scale Simulations of Nuclide Signals Responding to Rock Rupture.

A crack is usually conceptualized as two parallel plates separated with an aperture *b* = 2*b*_f_ and length *l* and bounded by the rock matrix (*SI Appendix*, Fig. S4). Under the current scenario where rock rupture is simplified to single-crack growth, the crack length *l* equals the signal transmission path length *L*. Here, we consider all possible sources and sinks for nuclide signals (*SI Appendix*, Fig. S4), including 1) *α*-recoil from the parent in the matrix, 2) parent-production from the surface coating, 3) adsorption to and 4) desorption from the surface, 5) decay loss in water, 6) parent-decay input in the crack domain, 7) precipitation, and 8) dissolution input from the matrix.

The transport of fluid-borne nuclides can be modeled using the advection–diffusion equations with appropriate wall boundary conditions:

[14a] $$\begin{array}{c} \frac{\partial C_{i}}{\partial t}=D\nabla^{2}C_{i}-\nabla \cdot \left(uC_{i}\right)\;\\ \text{+}\;\left[P_{r,i}-\lambda_{i}C_{i}+\left(f\lambda_{p,i}\frac{C_{sc,p,i}}{b_{f}}+\lambda_{p,i}C_{p,i}\right)+P_{w,i}-F_{i}C_{i}\right], \end{array}$$

[14b] $$\left(-D\nabla C_{i}+uC_{i}\right){|}_{\partial \Omega }=-b_{\text{f}}k_{1}C_{i}+k_{-1}C_{\mathrm{sc},i},$$

where *t* (s) is time, *D* (m^2^⋅s^−1^) is molecular diffusion coefficient, and **u** (m⋅s^−1^) is velocity vector of fluid carrier; *C_i_* and *C*_p,_*_i_* (mol⋅m^−3^) are bulk concentrations of target and parent nuclides in the crack domain, respectively, *C*_sc,_*_i_* and *C*_sc,p,_*_i_* (mol⋅m^−2^) are surface concentration of target and parent nuclides at surface coating, respectively; *P*_r,_*_i_* and *P*_w,_*_i_* (mol⋅m^−3^⋅s^−1^) are *α*-recoil and weathering rates, respectively; *λ_i_* and *λ*_p,_*_i_* (s^−1^) are decay constants of the target and parent nuclides, respectively, and *F_i_* (s^−1^) is precipitation rate; *f* (0 ∼ 1) is friction of target nuclide released in water by decay of its parent at surface coating; *k*_1_ and *k*_−1_ are desorption and adsorption coefficients, respectively, and ∂Ω refers to the wall boundary. The recoil is applied as a boundary condition: the volumetric recoil rate *P*_r_ [atom⋅L^−3^⋅T^−1^] is converted into an areal rate *p*_r_ [atom⋅L^−2^⋅T^−1^] by multiplying by *b*. Adsorption/desorption are implemented via Eq. **14b**. An analytical solution for key variable *C*_sc,_*_i_* is provided in *SI Appendix, Text S2*. Fluid flow is governed by the Navier–Stokes and continuity equations. Combining these with Eqs. **14a** and **14b** yields a pore-scale model for nuclide transport in rupture cracks.

We simulate fluid flow and nuclide transport by solving Eqs. **14a** and **14b** and Navier–Stokes equations. For flow, a constant inflow rate and zero pressure (outlet) are applied, with no-slip conditions on walls. For transport, fresh water (zero concentration) enters the inlet, an open boundary (∂*C_i_*/∂**n** = 0) is used at the outlet, and initial concentrations are zero everywhere, representing signal evolution in a newly formed crack without external sources. We consider two representative decay chains: ^226^Ra~^222^Rn and ^228^Ra~^224^Ra. Parameter values are introduced in *SI Appendix, Text S3* along with *SI Appendix*, Table S2). Both smooth (parallel-plate) and rough crack models are used, each with *l* = 0.1 m and mean aperture *b* = 1 mm (*SI Appendix*, Fig. S8). This aperture size captures essential flow and transport processes in fractured rocks (58, 70–72). The rough crack geometry comes from a split granite sample from Beishan, China, a candidate site for nuclear waste disposal (73), reconstructed via X-ray CT at 0.054 mm resolution (74).

Two key rock rupture states are simulated: crack dilation (varying *b*, implemented via changing *Zh*) and crack propagation (varying *S*, implemented via changing *P*_r_). Simulations run until steady state for the parent–daughter nuclide pairs. All simulations use COMSOL Multiphysics® with Lagrange quadrilateral/triangular elements and mesh refinement near boundaries (*SI Appendix*, Fig. S8). Models contain ∼10^6^ elements. Simulations run on a 96-core, 1 TB RAM workstation, taking 6 h to 5 wk per case (single chain, specific *Zh* and *P*_r_), totaling 6 mo and ~20 TB of results. Activity *A* (dpm/L) is used instead of concentration *C* for broader applicability, converted via *A_i_* = *C_i_*/*λ_i_*.

### Section 5. Examination and Calibration of Constitutive Relations.

To assess the established constitutive relations (Eqs. **10** and **13**), we test them under realistic and complex conditions. Nuclide signals during rupture fundamentally reflect dynamic changes in crack geometry and flow conditions, influenced by multiple physicochemical processes within developing cracks. Using high-resolution pore-scale simulations (*Section 4*), we examine the physical basis and practical applicability of the constitutive relations.

We perform pore-scale simulations of nuclide signals within single cracks (crack models illustrated in *SI Appendix*, Fig. S8) under key rupture states, covering different nuclide pairs (^226^Ra~^222^Rn and ^228^Ra~^224^Ra), crack properties (from smooth to rough), and flow regimes (from Poiseuille flow to heterogeneous velocity fields). Radium (^228^Ra, ^226^Ra, ^224^Ra) and radon (^222^Rn) are selected because they are ideal for tracing rupture due to their high *α*-recoil rates and mobility in fluid–rock systems (26). The simulations incorporate all sources and sinks affecting nuclide signals (Fig. 1*B*). For the simulation scheme, the crack dilation is represented by varying *Zh*, and the crack propagation by adjusting *P*_r_. Our simulations successfully reproduce the evolutionary pathways of nuclide signals induced by rupture (*SI Appendix*, Figs. S9 and S10), incorporating coevolving parent–daughter decay chains, comprehensive physicochemical processes, and realistic flow regimes in the crack. Results also align closely with analytical solutions (Fig. 3*B*).

Crucially, the simulations confirm that *Zh* and *P*_r_ govern the equilibrium signal *A*_eq_ (*SI Appendix*, Fig. S11*A*) and characteristic signal *A*_max_ (*SI Appendix*, Fig. S11*B*), respectively, as well described by constitutive Eqs. **10** and **13** (or Eq. **11**). Specifically, during crack dilation, *A*_eq_ follows a logistic function of *Zh* (Eq. **10**), with a sharp transition at mid-*Zh* values. This transition is particularly important for diagnostic application as it exhibits high sensitivity of *A*_eq_ to dilation-induced *Zh* (or *b*) changes. Different nuclides exhibit varying transition behaviors: while ^226^Ra~^222^Rn pair responds almost identically to *Zh*, ^224^Ra shows a broader and more gradual transition than its parent ^228^Ra (*SI Appendix*, Fig. S11*A*; slope values *κ* in *SI Appendix*, Table S3). ^222^Rn has the widest transition zone, enhancing its practical utility. The different sensitivities among these nuclides mainly stem from the significant differences in their half-lives as well as the variations in adsorption and desorption processes.

During propagation, *A*_max_ scales linearly with *P*_r_ across all signals (Eq. **11**), as depicted in *SI Appendix*, Fig. S11*B*. ^222^Rn shows the highest sensitivity (maximum slope Λ), followed by radium nuclides with lower Λ values (see *SI Appendix*, Table S4 for values of Λ), mainly due to additional sorption losses affecting radium. Thus, ^222^Rn is more effective for detecting crack propagation. Among radium nuclides, low sensitivities are ascribed to the freshwater setting with high adsorption–desorption coefficient (*k*_1_/*k*_−1_ = 1,000, *SI Appendix, Text S3*). However, when in saline water or high temperature settings (*k*_1_/*k*_−1_ = 1) (75, 76), ^224^Ra can acquire sufficiently high sensitivity to crack propagation with Λ = 0.7 (*SI Appendix*, Table S4) that could be feasibly detected by radium-specific detectors (e.g., RaDeCC, gamma spectrometry) (77, 78). Therefore, ^224^Ra can be practically employed to track crack propagation in saline or high-temperature contexts, e.g., brine intrusion, geothermal exploitation, and volcanic settings (9, 64, 75, 76).

Geometric heterogeneity of cracks minimally affects the performance of constitutive relations, but this situation would still require calibrating key parameters. Specifically, roughness slightly promotes sensitivity of *A*_eq_ to *Zh* (reflected by marginally higher *κ* listed in *SI Appendix*, Table S3), but it has little impact on the transition zone width (*SI Appendix*, Fig. S12). Meanwhile, roughness increases *A*_max_ by enhancing recoil input via increasing *S*_e_. Further analysis reveals that the enhancement ratios of *A*_max_ (1.08 for ^226^Ra and 1.04 for ^222^Rn; *SI Appendix*, Table S3) are broadly consistent with the surface area ratio (1.14) between rough and smooth cracks. This agreement provides quantitative evidence for the contribution of crack surface area to nuclide genesis. Moreover, surface roughness modestly impacts the *A*_max_ ∼ *P*_r_ relation by altering flow regimes and *S*_e_, thereby modulating Λ (*SI Appendix*, Table S4). Hence, the presence of crack roughness facilitates diagnosis by amplifying signal intensity and sensitivity to rock rupture.

## Supplementary Material

Appendix 01 (PDF)

## Data Availability

Previously published data were used for this work (2, 20). Other data are included in the article and/or *SI Appendix*.
