# Supplementary material for: Probing rock rupture with naturally occurring nuclide signals
Source: Proc Natl Acad Sci U S A. 2026 Apr 9;123(15):e2602434123. doi: 10.1073/pnas.2602434123 (PMC13079392; doi:10.1073/pnas.2602434123)
Supplement: Supplementary file 1 — Appendix 01 (PDF) [file pnas.2602434123.sapp.pdf]

Supporting Information for

Probing rock rupture with naturally occurring nuclide signals

Contributed by Michael Manga

Jia-Qing Zhou[a,b,c]†, Rong Mao[b,d]†, Xin Luo[b,*], M. Bayani Cardenas[e], Yi-Feng Chen[a,c,*], Fu-Shuo Gan[f], Chuang-Bing Zhou[a,c], Changdong Li[f], Huiming Tang[f], Ran Hu[a,c], Zhibing Yang[a,c], Michael Manga[g,*]
[a]State Key Laboratory of Water Resources Engineering and Management, Wuhan University; Wuhan, 430072, China.
[b]Laboratory for Sea Space Agent, Department of Earth and Planetary Sciences, The University of Hong Kong; Hong Kong, 999077, China.
[c]Key Laboratory of Rock Mechanics in Hydraulic Structural Engineering of the Ministry of Education, Wuhan University; Wuhan, 430072, China.
[d]Center for Natural Resources, Department of Civil and Environmental Engineering, New Jersey Institute of Technology; Newark, NY, 07102, USA.
[e]Department of Earth and Planetary Sciences, The University of Texas at Austin; Austin, TX, 787812, USA.
[f]Faculty of Engineering, China University of Geosciences; Wuhan, 430074, China
[g]Department of Earth and Planetary Science, University of California, Berkeley; Berkeley, CA, 94720, USA.

* Corresponding author. Xin Luo, Yi-Feng Chen, and Michael Manga
Email: xinluo@hku.hk; csyfchen@whu.edu.cn; mmanga@berkeley.edu
†These authors contributed equally to this work.

This PDF file includes:

Supporting text: Text S1-S5
Figures S1 to S13
Tables S1 to S8
SI References

Supporting Information Text

Text S1. Analytical solutions of the governing equations of radon transmission

Eq. 3 in M&M serves as the governing equation for radon transport during rock rupture. To obtain a closed-form solution, the following initial and boundary conditions are adopted: a zero ^{222}Rn concentration at the inlet, a free outflow boundary at the outlet, and a zero initial concentration throughout the domain:

$$C(0,t)=0,\quad \frac{\partial C(L,t)}{\partial x}=0 \qquad \text{[S1a]}$$

$$C(x,0)=0 \qquad \text{[S1b]}$$

These initial and boundary conditions represent the concentration evolution of radon within the newly-formed rock crack in the absence of any external sources.

The analytical solution of Eq. 3 along with initial-boundary conditions (Eq. S1) can be obtained using the method of separation of variables (1).

To separate the influence of advection on radon concentration from the influence of other processes, we assume:

$$C(x,t)=E(x,t)U(x,t) \qquad \text{[S2]}$$

where $E(x, t)$ mainly quantifies the influence of advection and decay on concentration, and $U(x, t)$ mainly quantifies the influence of diffusion and production on concentration.

Substituting Eq. S2 into the governing equation (Eq. 3) and rearranging it:

$$\frac{\partial U}{\partial t}=D\frac{\partial^2 U}{\partial x^2}+\left(\frac{2D}{E}\frac{\partial E}{\partial x}-\varpi L\right)\frac{\partial U}{\partial x}+\left(\frac{D}{E}\frac{\partial^2 E}{\partial x^2}-\frac{\varpi L}{E}\frac{\partial E}{\partial x}-\frac{1}{E}\frac{\partial E}{\partial t}-\lambda\right)U+\frac{P_{\mathrm{r}}}{E} \qquad \text{[S3]}$$

Based on the uniqueness principle of parabolic equation solutions, we decompose Eq. S3 into the following three equations:

$$\frac{2D}{E}\frac{\partial E}{\partial x}-\varpi L=0 \qquad \text{[S4a]}$$

$$\frac{D}{E}\frac{\partial^2 E}{\partial x^2}-\frac{\varpi L}{E}\frac{\partial E}{\partial x}-\frac{1}{E}\frac{\partial E}{\partial t}-\lambda=0 \qquad \text{[S4b]}$$

$$\frac{\partial U}{\partial t}=D\frac{\partial^2 U}{\partial x^2}+\frac{P_{\mathrm{r}}}{E} \qquad \text{[S4c]}$$

By solving Eqs. S4a and S4b, the solution for $E(x, t)$ is

$$E(x,t)=e^{\varpi Lx/2D}\cdot e^{-\left(\varpi^2L^2/4D+\lambda\right)t} \qquad \text{[S5]}$$

Substituting Eq. S5 into Eq. S4c, the governing equation for $U(x, t)$ is rearranged as

$$\frac{\partial U}{\partial t}=D\frac{\partial^2 U}{\partial x^2}+P_{\mathrm{r}}\cdot e^{-\varpi Lx/2D}\cdot e^{\left(\varpi^2L^2/4D+\lambda\right)t} \qquad \text{[S6]}$$

The initial and boundary conditions of Eq. S6 are obtained by substituting Eqs. S2 and S5 into the initial and boundary conditions of C (Eq. S1).

$$U(x,0)=0 \qquad \text{[S7a]}$$

$$U(0,t)=0,\quad \frac{\varpi L}{2D}U(L,t)+\left.\frac{\partial U}{\partial x}\right|_{x=L}=0 \qquad \text{[S7b]}$$

The governing equation of $U(x, t)$ (Eq. S6) is a parabolic partial differential equation, which is solved by the method of separation of variables and the method of eigenfunction expansion (1):

$$U(x,t)=\sum_{n=1}^{\infty}X_n(x)T_n(t) \qquad \text{[S8]}$$

where $X_n(x)$ is only a function of the spatial coordinate, and $T_n(t)$ is only a function of time. $X_n(x)$ is solved from the homogeneous equation of Eq. S6:

$$\frac{\partial U}{\partial t}=D\frac{\partial^2 U}{\partial x^2} \qquad \text{[S9]}$$

Substituting Eq. S8 into Eq. S9 yields

$$\frac{1}{X_n}\frac{\partial^2 X_n}{\partial x^2}=\frac{1}{D}\cdot\frac{1}{T_n}\cdot\frac{\partial T_n}{\partial t}=\gamma \quad \text{[S10]}$$

where γ is the eigenvalue of Eq. S10. Rearrange Eq. S10,

$$\frac{\partial^2 X_n}{\partial x^2}=\gamma X_n \quad \text{[S11a]}$$

$$\frac{\partial T_n}{\partial t}=\gamma D T_n \quad \text{[S11b]}$$

Substituting Eq. S8 into Eq. S7 yields the boundary conditions for $X_n(x)$ and $T_n(t)$

$$X_n\big|_{x=0}=0,\ \frac{\varpi L}{2D}X_n\big|_{x=L}+\frac{\partial X_n}{\partial x}\bigg|_{x=L}=0,\ T_n\big|_{t=0}=0 \quad \text{[S12]}$$

where $X_n(x)$ can be directly solved from Eqs. S11a and S12

$$X_n(x)=\sin\left(xk_n\right) \quad \text{[S13]}$$

and k_n is the solution to

$$\frac{\varpi L}{2D}\tan\left(Lk_n\right)=-k_n \quad \text{[S14]}$$

The value of Lk_n is shown in Fig. S5, which is the intersection point between the curve plotted by $y = \varpi L^2/2D\tan(x)$ and the line plotted by $y = -x$.

Substituting Eq. S13 into Eq. S10, the solution for $U(x, t)$ becomes

$$U(x,t)=\sum_{n=1}^{\infty}T_n(t)\sin\left(xk_n\right) \quad \text{[S15]}$$

Substituting Eq. S15 into Eq. S6 yields

$$\sum_{n=1}^{\infty}\frac{T_n(t)}{\partial t}\sin\left(xk_n\right)=-\sum_{n=1}^{\infty}D\cdot k_n^2\sin\left(xk_n\right)\cdot T_n+P_{\mathrm{r}}\cdot e^{-\varpi Lx/2D}\cdot e^{\left(\varpi^2L^2/4D+\lambda\right)t} \quad \text{[S16]}$$

which upon rearrangement becomes

$$\sum_{n=1}^{\infty}\left(\frac{T_n(t)}{\partial t}+Dk_n^2T_n\right)\sin\left(xk_n\right)=P_{\mathrm{r}}\cdot e^{-\varpi Lx/2D}\cdot e^{\left(\varpi^2L^2/4D+\lambda\right)t} \quad \text{[S17]}$$

Multiplying both sides by $\sin(xk_n)$, integrating over the interval from 0 to L, and using the orthogonality of the eigenfunction $\sin(xk_n)$ (see Eq. S18 below), Eq. S17 can be simplified to

$$\int_0^L\left[\sin\left(xk_{n1}\right)\cdot\sin\left(xk_{n2}\right)\right]\mathrm{d}x=\begin{cases}L_n, & \text{if } k_{n1}=k_{n2},\ \text{it is a constant}\\ 0, & \text{if } k_{n1}\neq k_{n2}\end{cases} \quad \text{[S18]}$$

We obtain the governing equation of $T_n(t)$:

$$\left(\frac{\partial T_n(t)}{\partial t}+Dk_n^2T_n\right)\int_0^L\sin^2\left(xk_n\right)\mathrm{d}x=\int_0^L P_{\mathrm{r}}\cdot e^{-\varpi Lx/2D}\cdot e^{\left(\varpi^2L^2/4D+\lambda\right)t}\cdot\sin\left(xk_n\right)\mathrm{d}x \quad \text{[S19]}$$

Eq. S19 is a nonhomogeneous linear first-order ordinary differential equation. The easiest method to solve it is to multiply it by an integrating factor $e^{Dk_n^2t}$. Thus,

$$e^{Dk_n^2t}\left(\frac{\partial T_n(t)}{\partial t}+Dk_n^2T_n\right)\int_0^L\sin^2\left(xk_n\right)\mathrm{d}x=e^{Dk_n^2t}\int_0^L P_{\mathrm{r}}\cdot e^{-\varpi Lx/2D}\cdot e^{\left(\varpi^2L^2/4D+\lambda\right)t}\cdot\sin\left(xk_n\right)\mathrm{d}x \quad \text{[S20]}$$

Rearranging Eq. S20

$$\frac{\partial T_n(t)e^{Dk_n^2t}}{\partial t}=\frac{e^{Dk_n^2t}}{\int_0^L\sin^2\left(xk_n\right)\mathrm{d}x}\int_0^L P_{\mathrm{r}}\cdot e^{-\varpi Lx/2D}\cdot e^{\left(\varpi^2L^2/4D+\lambda\right)t}\cdot\sin\left(xk_n\right)\mathrm{d}x \quad \text{[S21]}$$

and integrating Eq. S21 from 0 to t yields

$$T_n(t)e^{Dk_n^2t}-T_n(0)=\frac{1}{\int_0^L\sin^2\left(xk_n\right)\mathrm{d}x}\int_0^t e^{Dk_n^2\tau}\left(\int_0^L P_{\mathrm{r}}\cdot e^{-\varpi Lx/2D}\cdot e^{\left(\varpi^2L^2/4D+\lambda\right)\tau}\cdot\sin\left(xk_n\right)\mathrm{d}x\right)\mathrm{d}\tau \quad \text{[S22]}$$

where $T_n(0)$ is determined by the initial condition, i.e., $T_n(0) = 0$. Therefore, the solution for $T_n(t)$ is

$$T_n(t)=\frac{P_{\mathrm{r}}}{\int_0^L \sin^2\left(xk_n\right)\mathrm{d}x}e^{-Dk_n^2t}\int_0^t e^{Dk_n^2\tau}\cdot e^{\left(\varpi^2L^2/4D+\lambda\right)\tau}\cdot\left(\int_0^L \sin\left(xk_n\right)\cdot e^{-\varpi Lx/2D}\mathrm{d}x\right)\mathrm{d}\tau \qquad \text{[S23]}$$

Eq. S23 can be simplified using Eq. S14

$$T_n(t)=\frac{P_{\mathrm{r}}}{\int_0^L \sin^2\left(xk_n\right)\mathrm{d}x}\cdot\frac{k_n}{\left(\varpi L/2D\right)^2+k_n^2}\cdot\frac{1}{Dk_n^2+\left(\varpi L\right)^2/4D+\lambda}\cdot\left(e^{\left(\varpi^2L^2/4D+\lambda\right)t}-e^{-Dk_n^2t}\right) \qquad \text{[S24]}$$

Finally, the solution for $U(x, t)$ is obtained by substituting the solutions of $T_n(t)$ (Eq. S24) and $X_n(x)$ (Eq. S13):

$$U\left(x,t\right)=\sum_{n=1}^{\infty}\frac{P_{\mathrm{r}}}{\int_0^L \sin^2\left(xk_n\right)\mathrm{d}x}\cdot\frac{k_n}{\left(\varpi L/2D\right)^2+k_n^2}\cdot\frac{1}{Dk_n^2+\left(\varpi L\right)^2/4D+\lambda}\cdot\left(e^{\left(\varpi^2L^2/4D+\lambda\right)t}-e^{-Dk_n^2t}\right)\sin\left(xk_n\right) \qquad \text{[S25]}$$

where the integral $\int_0^L \sin^2\left(xk_n\right)\mathrm{d}x$ can be determined from the solution of k_n (Eq. S14):

$$\int_0^L \sin^2\left(xk_n\right)\mathrm{d}x=\frac{L}{2}+\frac{D/\left(\varpi L\right)}{\left(2Dk_n/\varpi^2L^2\right)^2+1} \qquad \text{[S26]}$$

By combining the solutions of $A(x, t)$ and $U(x ,t)$, the solution of $C(x ,t)$ is obtained

$$C\left(x,t\right)=\frac{P_{\mathrm{r}}}{\lambda}g_1\left(x,t;D,\lambda,\varpi,L\right) \qquad \text{[S27a]}$$

$$g_1=e^{\varpi Lx/2D}\cdot\sum_{n=1}^{\infty}\frac{1}{\int_0^L \sin^2\left(xk_n\right)\mathrm{d}x}\cdot\frac{k_n}{\left(\varpi L/2D\right)^2+k_n^2}\cdot\frac{\lambda}{Dk_n^2+\left(\varpi L\right)^2/4D+\lambda}\cdot\left(1-e^{-\left(Dk_n^2+\varpi^2L^2/4D+\lambda\right)t}\right)\sin\left(xk_n\right) \qquad \text{[S27b]}$$

According to Eq. S27, the signal A (= $\lambda C(L, t)$) at the outlet ($x = L$) of the crack is:

$$A=\lambda P_{\mathrm{r}}g_2\left(t;D,\lambda,\varpi,L\right) \qquad \text{[S28a]}$$

$$g_2=e^{\varpi L^2/2D}\cdot\sum_{n=1}^{\infty}\frac{1}{\int_0^L \sin^2\left(xk_n\right)\mathrm{d}x}\cdot\frac{k_n}{\left(\varpi L/2D\right)^2+k_n^2}\cdot\frac{1}{Dk_n^2+\left(\varpi L\right)^2/4D+\lambda}\cdot\left(1-e^{-\left(Dk_n^2+\varpi^2L^2/4D+\lambda\right)t}\right)\sin\left(Lk_n\right) \qquad \text{[S28b]}$$

This solution quantifies the breakthrough curves (BTCs) at the outlet in response to changes in flow regime (ϖ) and radon recoil rate (P_{r}) within the crack.

When t approaches infinity, the equilibrium signal A_{eq} of the BTC can be derived from Eq. S28:

$$A_{\mathrm{eq}}=\lambda P_{\mathrm{r}}g_3\left(D,\lambda,\varpi,L\right) \qquad \text{[S29a]}$$

$$g_3=e^{\varpi L^2/2D}\cdot\sum_{n=1}^{\infty}\frac{1}{\int_0^L \sin^2\left(xk_n\right)\mathrm{d}x}\cdot\frac{k_n}{\left(\varpi L/2D\right)^2+k_n^2}\cdot\frac{1}{Dk_n^2+\left(\varpi L\right)^2/4D+\lambda}\sin\left(Lk_n\right) \qquad \text{[S29b]}$$

The equilibrium signal increases as ϖ decreases, and it reaches the maximum value (A_{max}) when ϖ vanishes:

$$A_{\mathrm{max}}=\lambda P_{\mathrm{r}}g_4\left(D,\lambda,L\right) \qquad \text{[S30a]}$$

$$g_4\left(D,\lambda,L\right)=\sum_{n=1}^{\infty}\frac{1}{\int_0^L \sin^2\left(xk_n\right)\mathrm{d}x}\cdot\frac{1}{k_n}\cdot\frac{1}{Dk_n^2+\lambda}\sin\left(Lk_n\right) \qquad \text{[S30b]}$$

It should be noted that for other reaction contributions (processes 3 – 8 shown in Fig. S4), Eq. S30 includes an additional term A_0 (as defined in constitutive Eq. 11) to characterize these contributions. In the current radon configuration, A_0 is equal to 0. Furthermore, according to Eq. S30, the slope defined by constitutive Eq. 11 can be expressed as:

$$\Lambda=\lambda g_4\left(D,\lambda,L\right)=\lambda\sum_{n=1}^{\infty}\frac{1}{\int_0^L \sin^2\left(xk_n\right)\mathrm{d}x}\cdot\frac{1}{k_n}\cdot\frac{1}{Dk_n^2+\lambda}\sin\left(Lk_n\right) \qquad \text{[S31]}$$

Text S2. Derivation of key variable $C_{\mathrm{sc},i}$ in adsorption/desorption boundary conditions

The source and sink terms of adsorption and desorption at crack surfaces are described by

Eq. 14b in the form of boundary conditions. The governing equation describing nuclide concentration $C_{sc,i}$ at the surface coating is given by

$$\frac{\partial C_{\mathrm{sc},i}}{\partial t} = b_{\mathrm{f}} k_1 C_i - k_{-1} C_{\mathrm{sc},i} - \lambda_i C_{\mathrm{sc},i} + (1-f)\lambda_{\mathrm{p},i} C_{\mathrm{sc,p},i} \qquad \text{[S32]}$$

By restricting Eq. S32 with the initial condition $C_{sc,i}$ (t = 0) = $C_{sc,i,0}$, the analytical solution of $C_{sc,i}$ is obtained,

$$C_{\mathrm{sc},i} = \frac{b_{\mathrm{f}} k_1 C_i + (1-f)\lambda_{\mathrm{p},i} C_{\mathrm{sc,p},i}}{k_{-1}+\lambda_i} - \left[\frac{b_{\mathrm{f}} k_1 C_i + (1-f)\lambda_{\mathrm{p},i} C_{\mathrm{sc,p},i}}{k_{-1}+\lambda_i} - C_{\mathrm{sc},i,0}\right] e^{-(k_{-1}+\lambda_i)t} \qquad \text{[S33]}$$

$C_{sc,i,0}$ is the initial surface concentration of the target nuclide at the surface coating. The analytical solution is incorporated into Eq. 14b, together serving as the boundary conditions applied to the crack surfaces, which represent the adsorption and desorption terms depicted in Fig. S4.

Text S3. Physicochemical parameter justifications for Ra/Rn genesis and transmission

The parameters used for pore-scale simulations are summarized in Table S1. Simulations are performed under standard conditions, i.e., a temperature of 298.15 K and standard atmospheric pressure of 1 bar. These conditions determine the values of water density, viscosity, and the diffusion coefficients for radium and radon. The half-lives and decay constants of the radioactive nuclides remain constant, regardless of their physical or chemical environment. For the parameters associated with the eight reaction processes shown in Fig. S4, the values vary among the nuclides ^{228}Ra, ^{226}Ra, ^{224}Ra, ^{222}Rn.

(1) Alpha recoil rate from rock matrix

The alpha recoil rate quantifies the input rate of nuclides from the rock matrix into water or air, driven by the alpha decay of their parent nuclides. This rate depends on several factors, including the energy released during alpha decay, fluid properties, pore geometry (shape and size), rock type, and the activity of the parent nuclide within the rock matrix.

Typical alpha recoil rates in porous media range from 1 to 1000 atom·L^{-1}·min^{-1} for ^{226}Ra and ^{222}Rn, and from 10^{2} to 10^{5} atom·L^{-1}·min^{-1} for ^{228}Ra and ^{224}Ra. In this study, we adapt values representative of weathered volcanic rock: 1300 atom·L^{-1}·min^{-1} for ^{226}Ra and ^{222}Rn and 5324.8 atom·L^{-1}·min^{-1} for ^{228}Ra and ^{224}Ra (2).

(2) Weathering rate of nuclides in rock matrix

The chemical weathering rates of radionuclides are determined by the product of the bulk rock weathering rate and the nuclide activity within the minerals. The bulk rock weathering rate is set at 800 mg·L^{-1}·year^{-1}, representing a typical value for volcanic rock under standard temperature conditions (298.15 K). The activities of ^{238}U and ^{232}Th in volcanic rock are assumed to be 2 dpm·g^{-1}. Within minerals, radionuclides belonging to the same decay chain are always under secular equilibrium. Therefore, the activity of ^{226}Ra is equal to that of ^{238}U (2 dpm·g^{-1}), and the activities of ^{224}Ra and ^{228}Ra are equal to that of ^{232}Th (2 dpm·g^{-1}).

(3) Precipitation rate of radium

The chemical precipitation rate of radium is governed by its chemical properties and is therefore always the same for all radium nuclides. In freshwater environments, this rate is estimated to be 10 yr^{-1} (2), and it may be higher in saline conditions. In this study, we adopt a precipitation rate of 10 yr^{-1}, representative of freshwater environments.

(4) Desorption and adsorption rates of radium

Desorption and adsorption are governed by the chemical properties of radium, and thus all radium nuclides have the same desorption and adsorption rates. These rates are strongly influenced by salinity and temperature, and their relationships have been quantified in the study by Ivanovich and Harmon (3). In this study, we adapt values representative of freshwater conditions: a desorption rate of 11 min^{-1} and an adsorption rate of 0.011 min^{-1}, indicating the high adsorption-desorption coefficient k_1/k_{-1} = 1,000. For reference, we additionally include two sets of simulations with k_1/k_{-1} of 100 and 1 (Tables S3 and S4) to investigate the effects of gradually changing aquatic environments into high-temperature or saline conditions.

(5) Initial surface concentration of nuclides

The adsorbed activities of different radium nuclides have been measured in numerous studies

and exhibit a wide range, typically between 1 and 100 dpm·g^{-1}, depending on the salinity of the environment. In this study, an initial adsorbed activity of 2 dpm·g^{-1} is used for ^{228}Ra, ^{224}Ra, and ^{226}Ra.

(6) Fraction factor for nuclide releases due to alpha recoil from surface coating

The fraction factor is primarily determined by the thickness of the surface coating and the energy of the alpha recoil. For a typical coating thickness of 100 nm and alpha recoil from radium, the fraction factor is estimated to be approximately 0.5.

Text S4. Laboratory experiment settings and data compilation

The diagnostic theory is first assessed with the laboratory experiments. We perform these tests by adopting the experimental results from a well-designed month-long triaxial mechanic experiments conducted by Girault et al. (4). This experiment's settings are consistent with our physical model of nuclide signals responding to the crack dilation and closure, and to rock rupture.

Briefly, we adapt the experiment settings and results from a cylindrical granitic rock sample (a leucogranite from Allaire (AL), from France) representative of upper crustal rocks. Before the triaxial mechanical experiments, this AL sample (length: 8 cm, diameter: 4 cm) is thermally treated at 575 °C to generate initial micro-crack networks. Experiments on the AL core are conducted under dry upper crustal conditions using a triaxial oil-medium cell. The cell allows independent control of confining pressure, axial stress, and pore pressure (argon), to meet the independent control of rock rupture states and flushing rates. The dilation and closure of the initial cracks and their propagations are implemented by the experimental protocol: (1) increase confining pressure from 10 to 250 MPa (isostatic loading); (2) unload confining pressure from 250 to 70 MPa (isostatic unloading); (3) consistently apply differential stress to ~ 400 MPa until rock failure (deviatoric loading); (4) consistently unload differential stress to 0 (deviatoric unloading). Notably, throughout the loading and unloading processes, the constant injection of argon under a pressure difference (1 MPa) is maintained, enabling the calculation of rock sample permeability. Meanwhile radon signal in argon outflow is measured every 10 minutes using an AlphaGUARD™ ionization chamber. Each stress state is maintained until radon signals reach equilibrium activities (A_{eq}, marked by green circles in Fig. 2A), spanning a duration around 2-3 days for each state.

The AL sample is a typical REV. We conceptualize it to be a parallel plate, with the length (L) equal to 8 cm, width (w) equal to 4 cm, and b to be determined based on the equivalence of permeability k following the cubic law (5, 6):

$$Q = -\frac{wbk}{\mu}\nabla p = -\frac{wb^3}{12\mu}\nabla p \quad \text{[S34]}$$

$$k = \frac{b^2}{12} \quad \text{[S35]}$$

With the obtained equivalent b, we calculate the Zh number, defined as log ($q/\lambda bL$), at different stress states. According to the monitoring dataset (measured permeability k, and recorded radon signal A_{eq}), we obtain a total 10 Zh and A_{eq} as presented in Fig. 4A and detailed in Table S5.

Text S5. Field scale experiment and data

The model is further applied to track bedrock rupturing dynamics at the field scale. Such observations are scarce and more challenging to interpret because, unlike the lab experiment, geometric properties are unknown and uncontrolled. One good example is the extensive investigation conducted by Trique et al. (7), who measured 3-year long radon signal dynamics and the associated mechanical parameters in the hillslope tunnel proximal to a reservoir impacted by large water level fluctuation during impounding and drawdown. The water level (1480~1555 m above sea level) is always lower than the tunnel altitude.

This site is located in the French Alps at the boundary between the Belledonne crystalline basement and Permo-Triassic sedimentary formations. A monitoring tunnel with poor ventilation near Roselend dam has been equipped since 1995 with three radon detectors (BARASOL), two long-period seismometers, and a microbarograph. These instruments continuously record radon signals, tilt, and environmental conditions.

Long-term data reveal recurrent radon bursts inside the tunnel. We infer that bursts are induced by bedrock crack propagation and/or connection events and identified 12 main bursts (green circles

in Fig. 2B), with each followed by a series of residual bursts. We treat the peak of each radon burst to be the approximation of maximum radon signal ($\hat{A}_{\mathrm{max}}$), as the monitors directly record the emanation from the bedrock surface governed by crack radon diffusion, with negligible advection impacts. The crack propagation and/or connection events are delineated by the tilt observation and water level fluctuations, and the apparent exposed surface area $\hat{S}_{\mathrm{e}}$ is approximately quantified by the second-order derivative of water level over a time interval of 14 days (7). We thus finally obtain a total 12 pairs of ($\hat{S}_{\mathrm{e}}$, $\hat{A}_{\mathrm{max}}$) for main bursts, and further 2 pairs of ($\hat{S}_{\mathrm{e}}$, $\hat{A}_{\mathrm{max}}$) for residual bursts (Fig. 4B and Table S7).

Supporting Information Figures

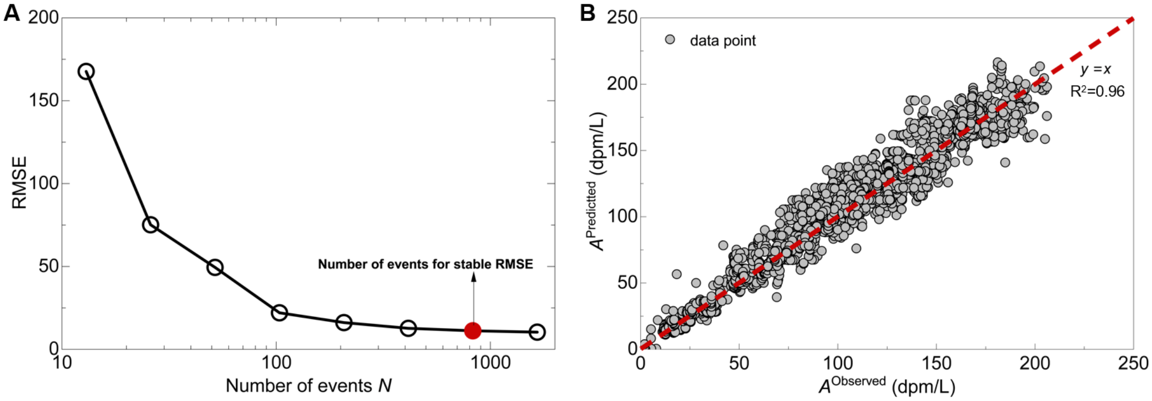

Fig. S1. Reconstruction error of radon signal time series observed from laboratory experiments conducted by Girault et al. (4). (A) Variation of root-mean-square error (RMSE) with increasing number of basic functions or micro-rupturing events (*N*). The curve plateaus after *N* = 832, suggesting that including additional events beyond this threshold yields negligible gains in fitting accuracy. (B) Error estimation of the reconstructed signal time series shown in Fig. 2A using *N* = 832. The determined parameters are listed in Table S1. The scatter plot demonstrates a high degree of agreement between predicted and observed signals, with data points tightly aligned along the diagonal 1:1 line with R^2 = 0.96.

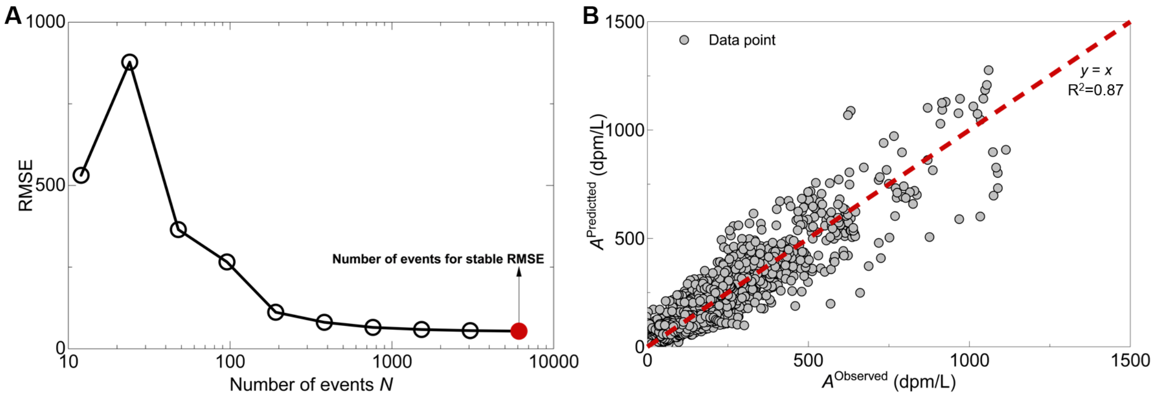

Fig. S2. Reconstruction error of radon signal time series monitored in the field setting by Trique et al. (7). (A) Variation of RMSE with increasing number of basic functions or micro-rupturing events (*N*). The curve plateaus after *N* = 6144, suggesting that including additional events beyond this threshold yields negligible gains in fitting accuracy. (B) Error estimation of the reconstructed signal time series shown in Fig. 2B using *N* = 6144. The determined parameters are listed in Table S1. The scatter plot demonstrates a high degree of agreement between predicted and observed signals, with data points tightly aligned along the 1:1 diagonal line with R^2 = 0.87.

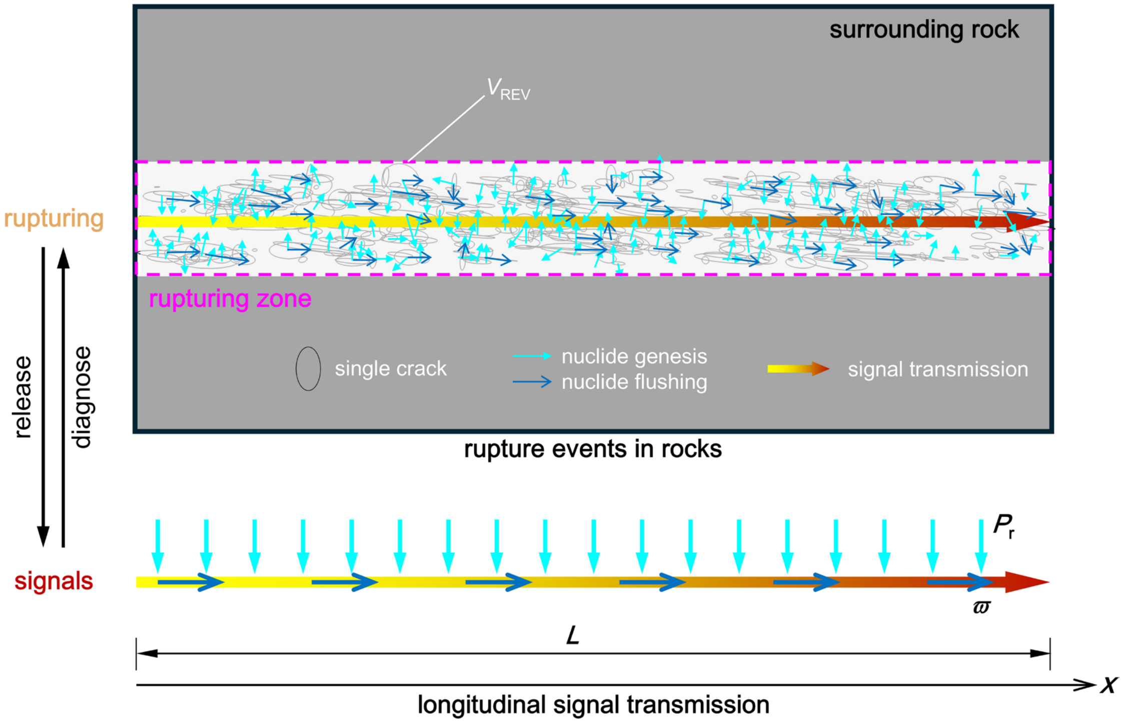

Fig. S3. One-dimensional (1D) model for nuclide signal transmission during rock rupturing. Within the 1D framework, aligned with both the longitudinal signal transmission axis (coinciding with the principal rupture direction), the signal transmissions released from stochastic and disordered rupture sequence (Fig. 3A) are upscaled through collapse into a homogeneous process characterized by equivalent physical quantities (i.e., recoil rate P_r and flushing rate ϖ). Specifically, increased crack intensity (quantified by rupture area S_e) manifests as enhanced uniform P_r input along the transmission path, while the enhanced nuclide flushing resulting from the expanded crack volume (quantified by rupture aperture b) is equivalent to elevated ϖ. The pink area in the figure represents the influence zone corresponding to the main rupture, and its delineated range is the representative elementary volume (V_{REV}) defined by Eq. 13.

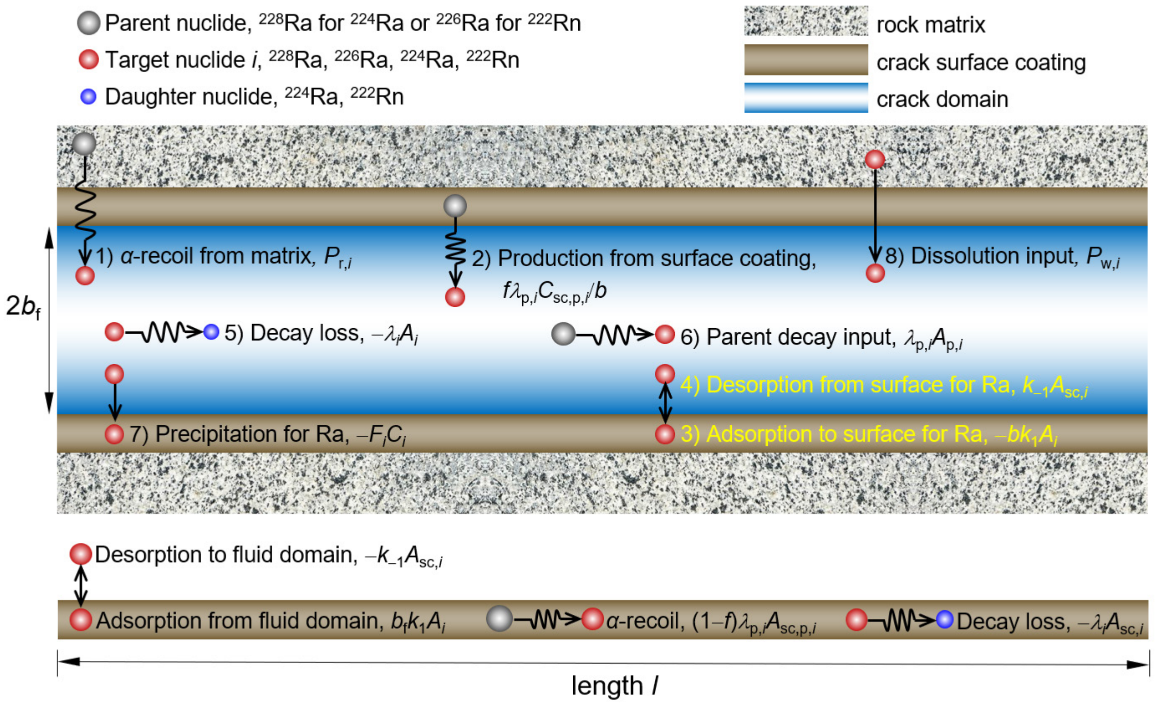

Fig. S4. Illustration of all possible physicochemical processes involved in representative naturally occurring nuclide pairs that are included in the mathematical formulations. These processes include: 1) α-recoil from the parent in the matrix, 2) parent-production from the surface coating, 3) adsorption to and 4) desorption from the surface, 5) decay loss in water, 6) parent-decay input in the crack domain, 7) precipitation, and 8) dissolution input from the matrix. The physical meaning of the variables in the figure can be found in Eq. 14.

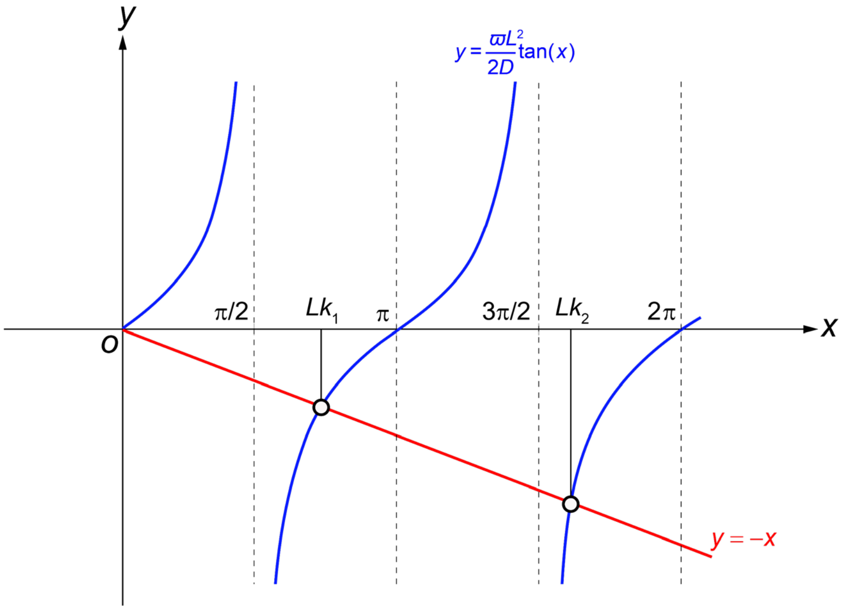

Fig. S5. Value of k_n (n = 1, 2, …) solved from Eq. S14. The value of Lk_n is the x-coordinate of the n-th intersection point between the curve of $y = \varpi L^2\tan(x)/2D$ and the line of $y = -x$.

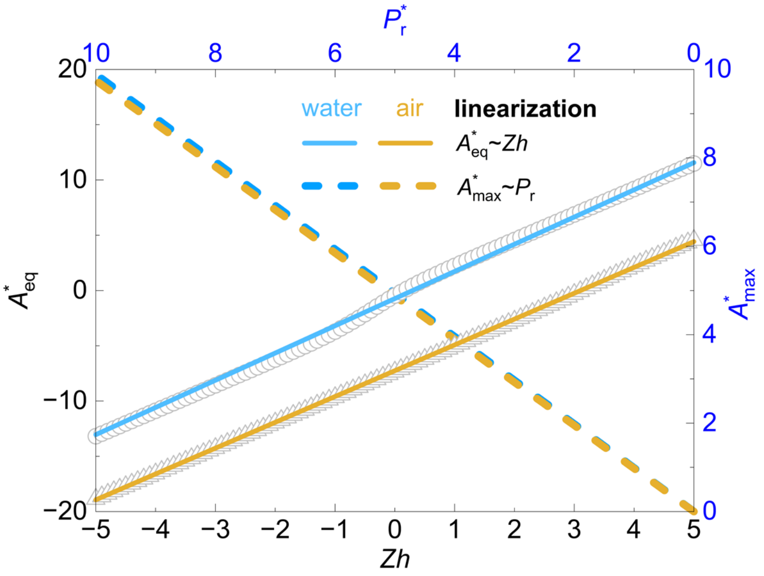

Fig. S6. Linearization of constitutive relations between characteristic nuclide signals (A_{eq} and A_{max}) and structure-related rupture parameters (Zh and P_r). A_{eq} and A_{max} are the equilibrium and maximum signals in the crack, respectively; P_r is the recoil rate, linearly proportional to rupture area; Zh is a newly defined dimensionless number, computed as $\log(\varpi/\lambda) = \log(q/\lambda bL)$, which is a mapping function of rupture aperture (b) and is also a normalization value of ϖ. ϖ and λ are flushing rate of the crack system and decay rate of the nuclide, respectively. q and L are the specific flow rate and signal transmission length in the rupture crack, respectively. The asterisk superscript in the figure denotes the dimensionless values of the variables. Specifically, P_r and A_{max} are normalized by the baseline values, while A_{eq} is normalized by A_{max} in the form of $A_{eq}^* = \ln(A_{max}/A_{eq}-1)$. Notably, A_{eq}^* quantifies the variation of crack dilation extent.

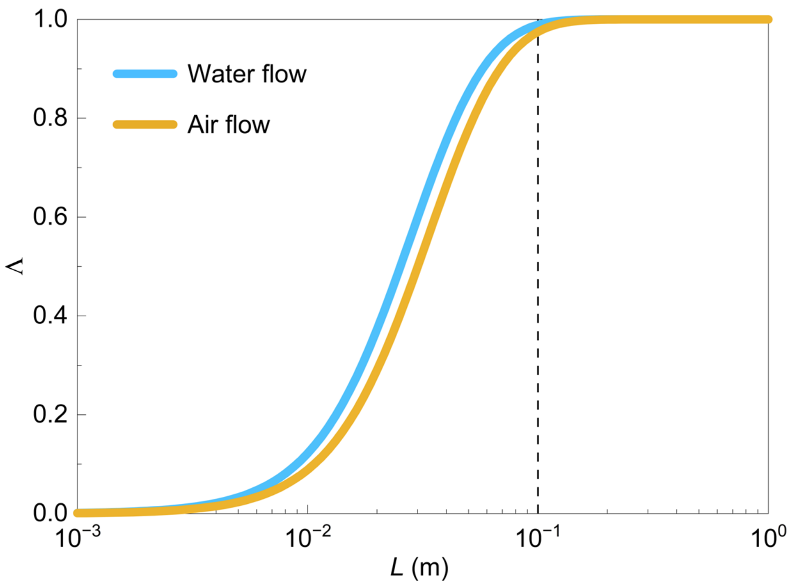

Fig. S7. Relationship between slope Λ and length *L*. Based on Eq. S31, Λ depends on signal transmission length *L* and diffusion coefficient *D*. The different curves between water and air flow result from the different diffusion coefficients.

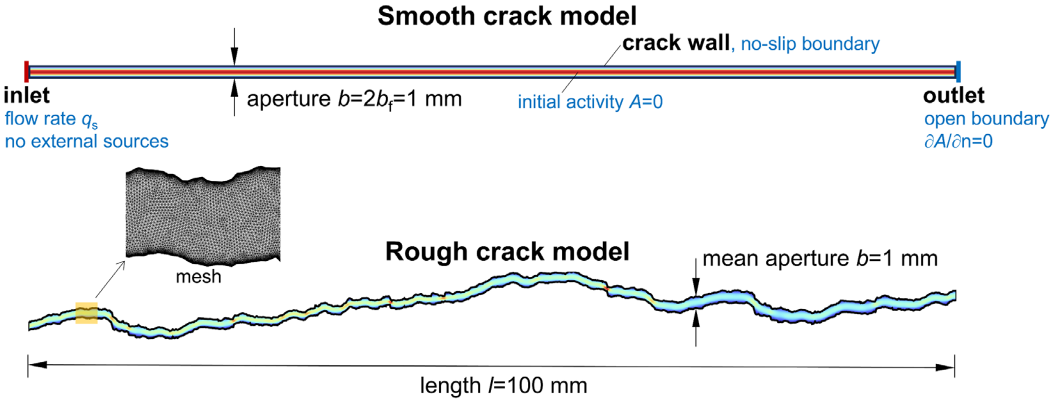

Fig. S8. Numerical models of nuclide signal transmission in ruptured cracks along with boundary and initial conditions. Smooth (i.e., idealized parallel-plates) and rough cracks (from split granite) have the same average aperture (b = 1 mm). The geometric morphology of the rough crack model is derived from a typical 2D cross-section reconstructed from CT scans of an artificially split granite crack (section 4 of M&M).

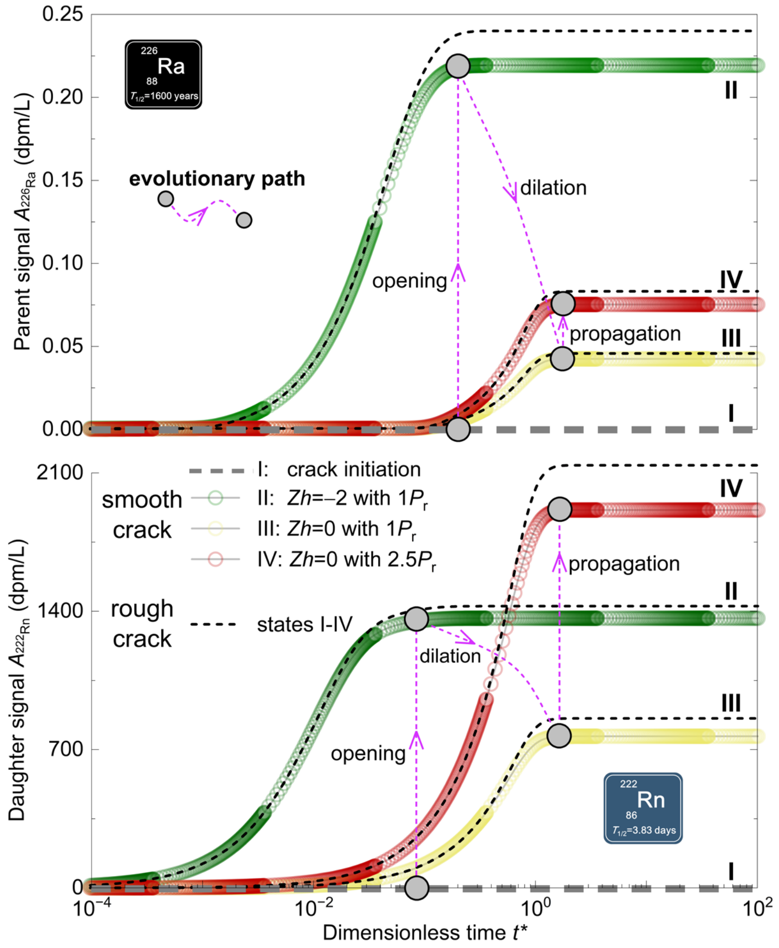

Fig. S9. Pore-scale simulation evolution of $^{226}Ra/^{222}Rn$ signals during rock rupture. The simulation accounts for parent-daughter inheritance relationships and incorporates all potential sources and sinks governing natural nuclide transport, as illustrated in Fig. S4. The pink curve marks the whole rock rupturing path, from crack initiation (state I), crack opening (state II), crack dilation (state III), to crack propagation (state IV), consistent with the physical model illustrated in Fig. 3A and analytical solutions present in Fig. 3B. To ensure consistency in parent-daughter pairs, the *Zh* values in the figure are calculated using the parent nuclide's decay rate. Notably, both smooth and rough cracks maintain identical average apertures (Fig. S8), to investigate the effect of surface roughness. The initial time point of all curves in the figure is defined relative to the initiation moment of a newly formed crack.

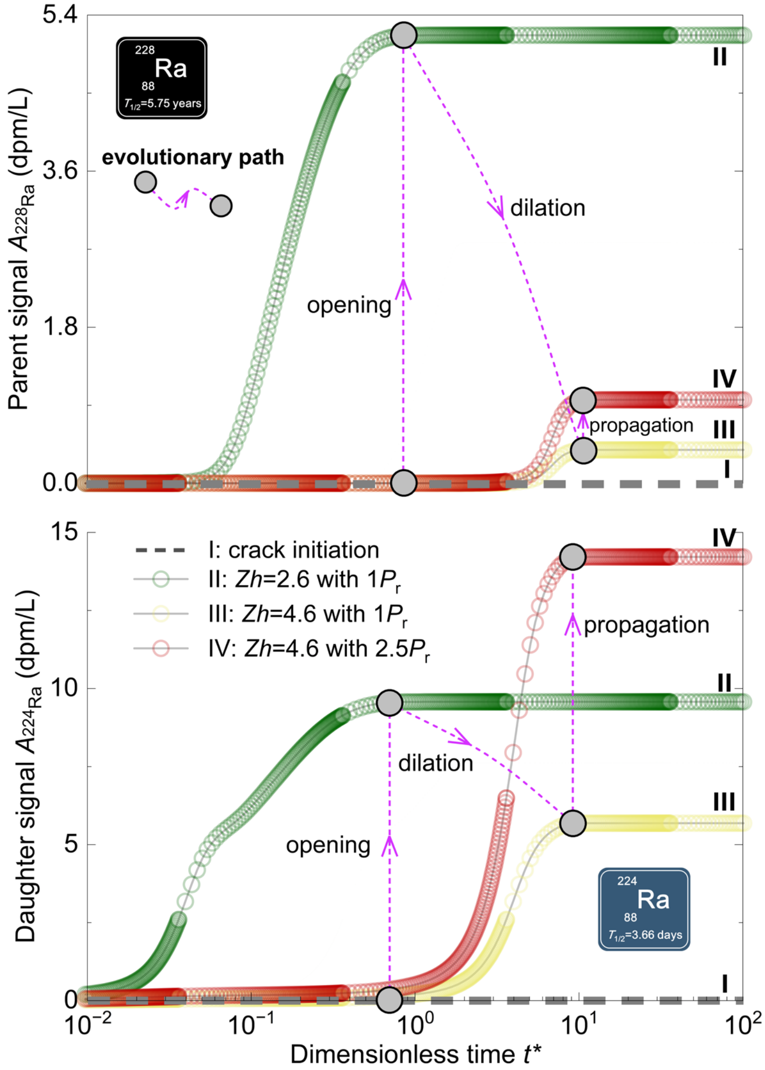

Fig. S10. Pore-scale simulation evolution of ^{228}Ra/^{224}Ra signals during rock rupture. The simulation accounts for parent-daughter inheritance relationships and incorporates all potential sources and sinks governing natural nuclide transport, as illustrated in Fig. S4. The pink curve marks the whole rock rupturing path, from intact rock (state I), crack initiation (state II), crack opening (state III), to crack propagation (state IV), consistent with the physical model illustrated in Fig. 3A and analytical solutions present in Fig. 3B. To ensure consistency in parent-daughter pairs, the *Zh* values in the figure are calculated using the parent nuclide's decay rate. All circle points represent results for the smooth crack (illustrated in Fig. S8). Notably, the initial time point of all curves in the figure is defined relative to the initiation moment of a newly formed crack.

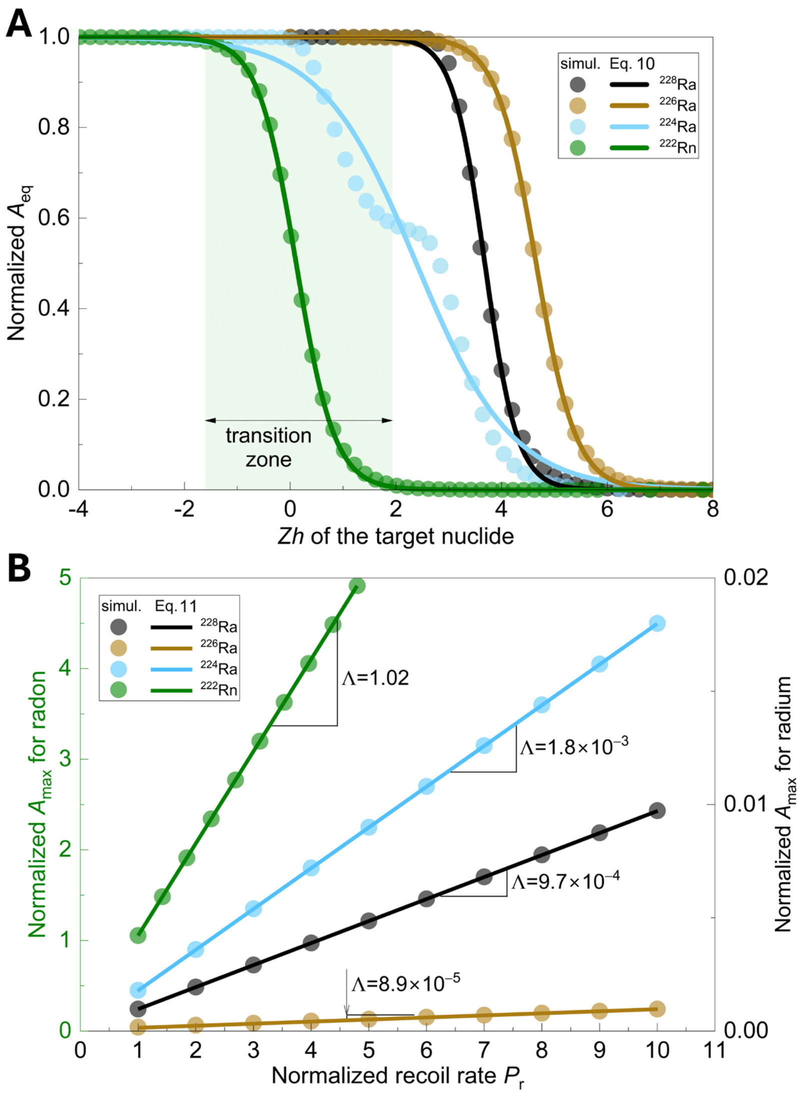

Fig. S11. Examination of constitutive relations between characteristic nuclide signals and geometric features of ruptures. **(A)** Constitutive Eq. 10 between A_{eq} and Zh (encapsulating rupture aperture information). The solid points represent pore-scale simulations, exhibiting a logistic trend well fitted by Eq. 10, indicated by the solid curves. The target nuclide's A_{eq} is normalized by its A_{max}. The transition zone (as exemplified by light green area for ^{222}Rn) represents the interval where A_{eq} varies rapidly with Zh, which holds greater practical significance for tracking rupture. **(B)** Constitutive Eq. 11 between A_{max} and P_r (encapsulating rupture area information). The solid points represent simulation results, exhibiting a strong linear trend (slope Λ) well fitted by Eq. 11, indicated by the solid lines. A_{max} and P_r are normalized by the baseline value. All fitting parameters are listed in Table S3 (for Eq. 10) and Table S4 (for Eq. 11).

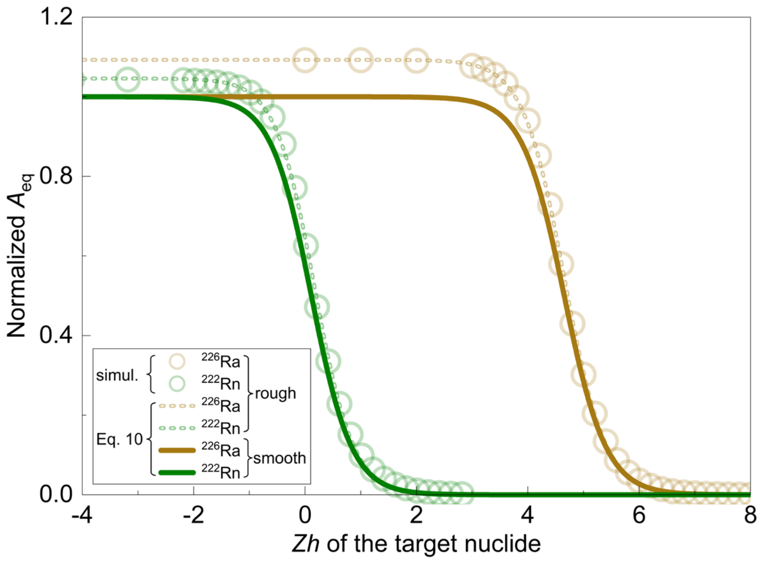

Fig. S12. Examination of the constitutive relation between A_{eq} and Zh (Eq. 10) for smooth and rough cracks, with multi-chain nuclides and reactions. All values of A_{eq} are normalized by A_{max} of the smooth crack. The circle points represent pore-scale simulation results, which can be well described by the constitutive Eq. 10 (illustrated by curves in the figure). All fitting parameters are listed in Table S3.

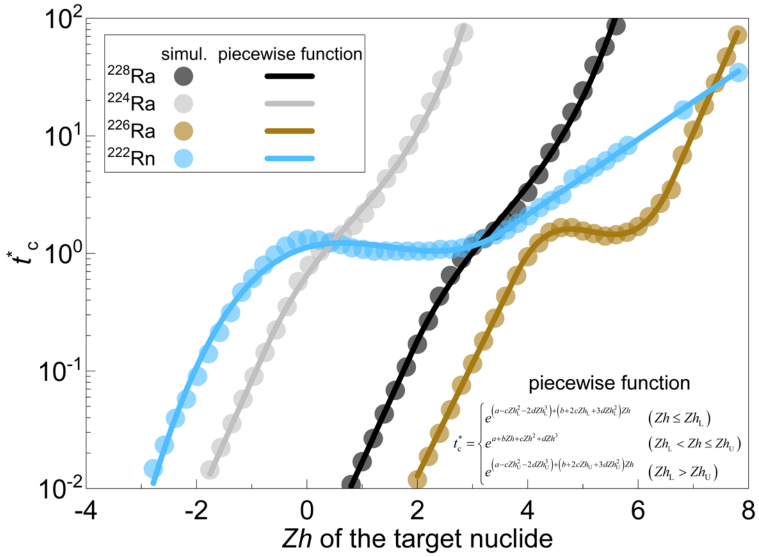

Fig. S13. Equilibrium time t_c for nuclide signals as an exponential function of dimensionless number *Zh* for various nuclides. Time *t* is non-dimensionalized as $t^* = tq/bL$. Solid points represent dimensionless t_c values determined from the simulation-derived *A~t* curves of the smooth crack (Figs. S9 and S10), which can be well described by the piecewise function noted in the figure. *a*, *b*, *c*, and *d* are the fitted coefficients, while Zh_L and Zh_U refer to *Zh* at the lower and upper segmentation points, respectively.

Supporting Information Tables

Table S1. Summary of optimized parameters for radon signal time series reconstruction.

Parameter	Statistic	Laboratory experiment	Field scale observation
χ_i	Range	22.46~1.99×10^9	5.53~2.49×10^7
	Mean	7.05×10^6	4.48×10^3
	Std. Dev.	8.76×10^7	3.22×10^5
γ_i	Range	3.19~166.87	14.2~2.59×10^9
	Mean	27.13	2.63×10^6
	Std. Dev.	11.21	6.17×10^7
τ_0^i [d]	Range	0.08~29.60	0.09~861.62
	Mean	16.89	430.67
	Std. Dev.	7.51	248.87
τ_{peak}^i [d]	Range	0.51~29.62	0.42~861.92
	Mean	16.97	430.96
	Std. Dev.	7.52	248.87
τ_{start}^i [d]	Range	0~29.51	0~860.92
	Mean	16.71	429.99
	Std. Dev.	7.50	248.86
τ_{end}^i [d]	Range	1.03~30.66	0.27~861.87
	Mean	17.67	430.94
	Std. Dev.	7.50	248.86
δ	/	1.77×10^4	3.98×10^5
φ	Range	0.40~0.90	0.40~0.90
	Mean	0.65	0.64
	Std. Dev.	0.07	0.07

Table S2. Physical parameter values for pore-scale direct numerical simulations.

Physical parameter	Unit	Parameter value or expression*	References
Water density, ρ	kg/m^3	1000	/
Rock density, ρ_{rock}	kg/m^3	2800	/
Dynamic viscosity of water, μ	Pa·s	8.91×10^{-4}	/
Boltzmann constant, k_B	J/K	1.38×10^{-23}	Newell et al. (8)
Atomic radius of radium, r_{Ra}	pm	283	Los Alamos National Laboratory
Atomic radius of radon, r_{Rn}	pm	220	
Absolute temperature, T	K	298.15	/
Diffusion coefficient of radium, D_{Ra}	m^2/s	$k_B T/6/\pi/\mu/r_{\mathrm{Ra}}$	Stokes-Einstein equation
Diffusion coefficient of radon, D_{Rn}	m^2/s	$k_B T/6/\pi/\mu/r_{\mathrm{Rn}}$	
α-recoil range, r	μm	0.05	Luo et al. (2)
Half-life of ^{228}Ra, t_{hf}^{228}	y	5.75	Porcelli (9)
Half-life of ^{226}Ra, t_{h}^{226}	y	1600	
Half-life of ^{224}Ra, t_{hf}^{224}	d	3.66	
Half-life of ^{222}Rn, t_{hf}^{222}	d	3.825	
Half-life of ^{232}Th (parent of ^{228}Ra), t_{hf}^{232}	y	1.41×10^{10}	
Half-life of ^{230}Th (parent of ^{226}Ra), t_{hf}^{230}	y	7.54×10^{4}	
Decay constant of ^{228}Ra, λ_{228}	1/s	$\log2/t_{\mathrm{hf}}^{228}$	/
Decay constant of ^{226}Ra, λ_{226}	1/s	$\log2/t_{\mathrm{hf}}^{226}$	/
Decay constant of ^{224}Ra, λ_{224}	1/s	$\log2/t_{\mathrm{hf}}^{224}$	/
Decay constant of ^{222}Rn, λ_{222}	1/s	$\log2/t_{\mathrm{hf}}^{222}$	/
Decay constant of ^{232}Th, λ_{232}	1/s	$\log2/t_{\mathrm{hf}}^{232}$	/
Decay constant of ^{230}Th, λ_{230}	1/s	$\log2/t_{\mathrm{hf}}^{230}$	/
Decay constant of ^{227}Ac, λ_{227}	1/s	$\log2/t_{\mathrm{hf}}^{227}$	/
α-recoil rate of ^{228}Ra, $P_{r,228}$	atom/L/min	$4.096P_{r,226}$	Ivanovich et al. (3)
α-recoil rate of ^{226}Ra, $P_{r,226}$	atom/L/min	1300	Luo et al. (2)

Surface α-recoil rate of ^{226}Ra, $p_{r,226}$	atom/m^2/min	$1300\times10^3\times1\times10^{-3}$	Luo et al. (2), 1 mm aperture
α-recoil rate of ^{224}Ra, $P_{r,224}$	atom/L/min	$P_{r,228}$	/
α-recoil rate of ^{222}Rn, $P_{r,222}$	atom/L/min	$P_{r,226}$	/
Chemical weathering rate of bulk rock, P_{rw}	mg/L/y	800	Luo et al. (2)
Activity concentration inside mineral lattice in fresh rocks, A^r_{U238}	dpm/g	2	
Weathering rate of ^{228}Ra, $P_{w,228}$	atom/L/min	$P_{rw}A^r_{U238}/\lambda_{228}$	/
Weathering rate of ^{226}Ra, $P_{w,226}$	atom/L/min	$P_{rw}A^r_{U238}/\lambda_{226}$	/
Weathering rate of ^{224}Ra, $P_{w,224}$	atom/L/min	$P_{rw}A^r_{U238}/\lambda_{224}$	/
Precipitation rate of radium, F_{Ra}	1/y	10	Luo et al. (2)
Friction factor, f	-	0.5	/
Desorption coefficient, k_{-1}	1/min	k_1/1000	Krishnaswami et al. (10)
Adsorption coefficient, k_1	1/min	11	
Activity concentration in absorption phase, $A^r_{Ra\_ab}$	dpm/g	2	Moore et al. (11)
Initial surface concentration of ^{228}Ra, $C_{sc,228,0}$	atom/m^2	$r\rho_{rock}A^r_{U238}/60/\lambda_{228}$	/
Initial surface concentration of ^{226}Ra, $C_{sc,226,0}$	atom/m^2	$r\rho_{rock}A^r_{U238}/60/\lambda_{226}$	/
Initial surface concentration of ^{224}Ra, $C_{sc,224,0}$	atom/m^2	$r\rho_{rock}A^r_{U238}/60/\lambda_{224}$	/

*Part of the expressions involves unit conversion, which strictly obeys the dimension consistency.

Table S3. Parameter calibration of constitutive Eq. 10 based on analytical solutions and pore-scale simulations.

Nuclides	Crack model	Eq. 10 for A_{eq}~Zh		
		A_{max} (dpm/L)	κ (-)	Zh_0 (-)
Parent ^{226}Ra	smooth	0.225	2.654	4.653
	rough	0.245	2.703	4.653
	sensitivity analysis of k_1/k_{-1} (=100)	/	/	/
	sensitivity analysis of k_1/k_{-1} (=1)	/	/	/
Daughter ^{222}Rn	analytical solutions (water)	1267.7	2.503	0.794
	analytical solutions (air)	1.248	2.338	7.262
	smooth	1370.5	2.752	0.114
	rough	1429.6	2.858	0.168
	smooth - sensitivity analysis of k_1/k_{-1} (=100)	/	/	/
	smooth - sensitivity analysis of k_1/k_{-1} (=1)	/	/	/
Parent ^{228}Ra	smooth	5.184	3.268	3.683
	smooth - sensitivity analysis of k_1/k_{-1} (=100)	/	/	/
	smooth - sensitivity analysis of k_1/k_{-1} (=1)	/	/	/
Daughter ^{224}Ra	smooth	9.587	1.104	2.389
	sensitivity analysis of k_1/k_{-1} (=100)	/	/	/
	sensitivity analysis of k_1/k_{-1} (=1)	/	/	/

Table S4. Parameter calibration of constitutive Eq. 11 based on analytical solutions and pore-scale simulations.

Nuclides	Crack model	Eq. 11 for A_{max}~P_r	
		Slope Λ (-)	Intercept A_0 (dpm·L^{-1})
Parent ^{226}Ra	smooth	8.9×10^{-5}	0.109
	rough	9.9×10^{-5}	0.116
	sensitivity analysis of k_1/k_{-1} (= 100)	9.6×10^{-5}	0.118
	sensitivity analysis of k_1/k_{-1} (= 1)	9.6×10^{-5}	0.119
Daughter ^{222}Rn	analytical solutions (water)	0.989	/
	analytical solutions (air)	0.975	/
	smooth	1.016	50.15
	rough	1.058	54.43
	smooth - sensitivity analysis of k_1/k_{-1} (= 100)	0.979	5.480
	smooth - sensitivity analysis of k_1/k_{-1} (= 1)	0.975	0.163
Parent ^{228}Ra	smooth	9.7×10^{-4}	4.252×10^{-3}
	smooth - sensitivity analysis of k_1/k_{-1} (= 100)	7.6×10^{-3}	3.366×10^{-2}
	smooth - sensitivity analysis of k_1/k_{-1} (= 1)	2.6×10^{-2}	0.114
Daughter ^{224}Ra	smooth	1.8×10^{-3}	3.548×10^{-3}
	sensitivity analysis of k_1/k_{-1} (= 100)	1.8×10^{-2}	2.303×10^{-2}
	sensitivity analysis of k_1/k_{-1} (= 1)	0.742	8.735×10^{-2}

Table S5. Dataset collected from month-long triaxial experiments conducted by Girault et al. (4).

Loading path	Stress (MPa)	A_{eq} (dmp·L^{-1})	Measured permeability (m^2)	Calculated aperture (μm)#	*Zh*
isostatic loading	10	13.6 (6.0~23.1)	5.90×10^{-18}	8.41×10^{-3}	9.40
	20	25.9 (8.5~38.9)	2.66×10^{-18}	5.65×10^{-3}	9.23
	50	48.2 (28.2~69.2)	1.16×10^{-18}	3.73×10^{-3}	9.05
	70	81.7 (57.9~103.9)	5.43×10^{-19}	2.55×10^{-3}	8.88
	100	103.3 (81.0~130.0)	3.31×10^{-19}	1.99×10^{-3}	8.77
	170	120.8 (99.5~145.8)	1.55×10^{-19}	1.36×10^{-3}	8.61
	250	125.0 (99.3~154.7)	4.41×10^{-20}	7.28×10^{-4}	8.34
isostatic unloading	70	171.1 (137.3~206.1)	1.83×10^{-19}	1.48×10^{-3}	8.65
deviatoric loading	300	162.4 (121.7~197.4)	1.66×10^{-19}	1.41×10^{-3}	8.62
	370	165.9 (134.4~196.0)	2.11×10^{-19}	1.59×10^{-3}	8.68
	450 (failure)	179.4 (137.0~212.2)	6.12×10^{-19}	2.71×10^{-3}	8.91
deviatoric unloading	220	97.1 (74.4~126.9)	8.00×10^{-19}	3.10×10^{-3}	8.97
	0	64.2 (48.5~80.8)	1.25×10^{-18}	3.87×10^{-3}	9.06

Equivalent aperture is calculated from the measured permeability according to the cubic law (5, 6).

Table S6. Parameter values of Eq. 1a for reproducing radon signal evolution during crack closing/opening in laboratory experiments (Fig. 4A).

Loading pattern	A_{max} (dpm/L)	κ (-)	Zh_0 (-)	R^2
Isostatic loading	130.84	5.96	8.98	0.995
Deviatoric unloading	130.84×142%[#]	20.00	9.00	0.814

[#] Percentage increase in A_{max} after rupturing adopts calibrated value reported by Girault et al. (4), rather than the fitted value.

Table S7. Dataset collected from 3-year long field observations reported by Trique et al. (7).

Main burst (MB)	Residual burst (RB)	Peak signal $\hat{A}_{\max}$ (dpm/L)*	Apparent rupture area ($\hat{S}_e$)**	Burst time (months)#
MB 1	/	441.1	0.13	1.0
MB 2	/	841.5	1.00	2.1
MB 3	RB 1 or MB3	1113.8	0.61	5.0
/	RB 2 after MB3	898.2	0.58	5.1
/	RB 3 after MB3	448.5	0.34	5.8
/	RB 4 after MB3	354.8	0.42	6.1
/	RB 5 after MB3	126.9	0.08	6.8
/	RB 6 after MB3	178.6	0.47	7.0
/	RB 7 after MB3	154.0	0.25	7.3
/	RB 8 after MB3	55.4	0.06	7.6
MB 4	RB 9 after MB3	303.1	0.21	8.5
/	RB 10 after MB3	215.6	0.16	9.6
MB 5	RB 11 after MB3	282.1	0.21	10.2
/	RB 12 after MB3	87.5	0.04	11.6
MB 6	RB 1 or MB6	499.0	0.32	11.9
/	RB 2 after MB6	227.9	0.13	12.2
/	RB 3 after MB6	99.8	0.08	12.9
/	RB 4 after MB6	174.9	0.10	13.1
MB 7	RB 5 after MB6	172.5	0.11	13.9
/	RB 6 after MB6	70.2	0.16	14.0
/	RB 7 after MB6	129.4	0.16	15.7
MB 8	/	649.3	0.50	16.3
MB 9	/	235.3	0.09	23.7
MB 10	/	531.0	0.36	25.7
MB 11	/	314.2	0.27	26.5
MB 12	/	449.7	0.16	28.0

* 10% temporal uncertainty of the radon monitoring device BARASOL (7, 12).

** $\hat{S}_e$ is expressed in arbitrary unit.

Starting date is October 22, 1995.

Table S8. Parameter values of Eq. 1b for reproducing radon signal evolution during crack propagation in the field observations (Fig. 4B).

Radon outburst	$\hat{S}_e$	A_0 (dpm/L)	R^2
MBs (between Oct. 1995 and Mar. 1998)	841.3	208.0	0.66
RBs after MB3 (between Mar. 1996 and Oct. 1996)	1279.5	0	0.86
RBs after MB6 (between Oct. 1996 and Feb. 1997)	1331.6	0	0.68

[#] Since the apparent rupture area ($\hat{S}_e$) is quantified in arbitrary unit, the slopes $\hat{\Gamma}$ presented in this table represent nominal values and are not directly equivalent to the slope parameter in Eq. 1b.

References

1. N. H. Asmar, *Partial differential equations with Fourier series and boundary value problems* (Courier Dover Publications, 2016).
2. S. Luo, T.-L. Ku, R. Roback, M. Murrell, T. L. McLing, In-situ radionuclide transport and preferential groundwater flows at INEEL (Idaho): decay-series disequilibrium studies. *Geochimica Et Cosmochimica Acta* **64**, 867–881 (2000).
3. M. Ivanovich, R. S. Harmon, Uranium-series disequilibrium: applications to earth, marine, and environmental sciences. 2. (1992).
4. F. Girault, A. Schubnel, É. Pili, Transient radon signals driven by fluid pressure pulse, micro-crack closure, and failure during granite deformation experiments. *Earth and Planetary Science Letters* **474**, 409–418 (2017).
5. R. W. Zimmerman, G. S. Bodvarsson, Hydraulic conductivity of rock fractures. *Transport in porous media* **23**, 1–30 (1996).
6. J. Q. Zhou, S. H. Hu, S. Fang, Y. F. Chen, C. B. Zhou, Nonlinear flow behavior at low Reynolds numbers through rough-walled fractures subjected to normal compressive loading. *International Journal of Rock Mechanics and Mining Sciences* **80**, 202–218 (2015).
7. M. Trique, P. Richon, F. Perrier, J. P. Avouac, J. C. Sabroux, Radon emanation and electric potential variations associated with transient deformation near reservoir lakes. *Nature* **399**, 137–141 (1999).
8. D. B. Newell, *et al.*, The CODATA 2017 values of h, e, k, and NA for the revision of the SI. *Metrologia* **55**, L13 (2018).
9. D. Porcelli, Investigating groundwater processes using U-and Th-series nuclides. *Radioactivity in the Environment* **13**, 105–153 (2008).
10. S. Krishnaswami, W. C. Graustein, K. K. Turekian, J. F. Dowd, Radium, thorium and radioactive lead isotopes in groundwaters: Application to the in situ determination of adsorption-desorption rate constants and retardation factors. *Water Resources Research* **18**, 1663–1675 (1982).
11. W. S. Moore, R. Arnold, Measurement of 223Ra and 224Ra in coastal waters using a delayed coincidence counter. *Journal of Geophysical Research: Oceans* **101**, 1321–1329 (1996).
12. H. Zafrir, Y. Ben Horin, U. Malik, C. Chemo, Z. Zalevsky, Novel determination of radon-222 velocity in deep subsurface rocks and the feasibility to using radon as an earthquake precursor. *JGR Solid Earth* **121**, 6346–6364 (2016).
